# Nursing Home-Sensitive Hospitalizations and the Relevance of Telemedicine: A Scoping Review

**DOI:** 10.3390/ijerph191912944

**Published:** 2022-10-10

**Authors:** Maria Paula Valk-Draad, Sabine Bohnet-Joschko

**Affiliations:** Chair of Health Care Management and Innovation, Faculty of Management, Economics, and Society, Witten/Herdecke University, Alfred-Herrhausen-Str. 50, 58448 Witten, Germany

**Keywords:** telemedicine, telehealth, nursing home, nursing home resident, full inpatient, long-term care, hospitalization, intervention, implementation science, scoping review

## Abstract

The aging of society is increasing the number of hospitalizations of nursing home residents. Telemedicine might help reduce the frequency of these potentially risk-associated hospitalizations. This scoping review looked for evidence of a change in the rate of hospitalization and, if mentioned, any cost savings and/or staff acceptance of the use of telemedicine in a nursing home setting. To identify available evidence, the electronic databases PubMed, Livivo, EBSCO and JSTOR were searched (without time or regional constraints) for comparative primary research studies on this topic in peer-reviewed journals. A total of 1127 articles were retrieved and 923 titles and abstracts were screened, with 16 studies published between 2001 and 2022 being included. Telemedicine consultation reduced the hospitalization of nursing home residents in 14/16 and care costs in 8/11 articles. Staff satisfaction was mentioned positively in five studies. Most studies used telemedicine involving medical diagnostic technologies (10), (electronic) health records (9), specialists (9) and specialized nursing staff (11). Few studies had a higher level of evidence: only one randomized clinical trial was included. There is the need for high credibility studies, using guidelines on protocol and reporting, to better understand the hindering and facilitating factors of telemedicine provision in the healthcare of nursing home residents.

## 1. Introduction

Hospital (re)admission from long-term care and post-acute care facilities is common and its risk factors might signify inadequate care processes or a mismatch between patient needs and facility resources [1,2,3,4,5]. As the proportion of those over 65-years old rises across Europe, Australia, New Zealand, North-America, the Middle East and Asiatic countries, the numbers of those in need of long-term care are also increasing and with them also the number of hospitalizations [6,7,8,9,10,11]. For the vulnerable population of nursing home residents (NHRs), who often suffer from comorbidity, it is particularly important to reduce hospitalizations, as these are often associated with nosocomial health risks [12,13,14,15,16]. Hospitalizing NHRs is to some extent inappropriate and preventable [2,4,17,18]. An estimated 35% of all hospitalizations due to so called nursing home-sensitive conditions (NHSCs) among nursing home residents (NHRs) could be prevented and be treated in the nursing home (NH) under optimal care conditions [2] or by combining multiple interventions to reduce hospitalizations of NHRs [19,20].

The quality of care in NHs is related to the ratio of nursing staff to NHRs, which is often described as inadequate for NHs [21]. This is related to high work loads, time constraints in accomplishing all nursing tasks and the risk of burnout among nursing health care aides and nurses, especially since the care needs of NHRs are highly complex, which in turn leads to even more work stress and additional risks for NHRs (e.g., falls, undetected infections, neglect or even abuse) [22,23,24]. Other factors related to the quality of care in the NHs are unmet needs regarding medical specialist utilization, especially in rural areas [25,26]. To improve care conditions and consequently reduce hospitalizations, certain changes and/or interventions are needed in NHs but also in health care systems. These alterations build on each other and often only become effective when they interlock [27,28,29,30]. Telemedicine is one of many possible interventions that could meet some of the requirements to improve quality of care for NHRs. These requirements concern more cross-sector cooperation, communication and partnerships. In case of understaffing, especially of advanced practice or registered nurses, there might be uncertainty in the case of a deterioration of a NHR’s health status and staff might not be able to provide the NHR with the care he/she needs. Telemedicine then offers NH nursing staff the possibility of easily accessible and rapid consultation with medical specialists in hospitals (e.g., geriatricians and emergency care departments), general physician practices, pharmacists, dentists or therapists (e.g., speech/physio-/ergotherapists) to alleviate any uncertainties regarding the necessary care needs of the NHR. Telemedicine consultation on the care needs of NHRs could take place in daily case conferencing between some of these professions and the NH staff [28,30,31] as well as NHRs and family caregivers. Cooperation agreements, including telemedicine services, could also be set up between these professionals and NHs to offer services for NHs in rural areas or for NHs with unmet needs.

There is high diversity in terms of the definitions and descriptions of telemedicine, the technologies included and the complexity and range of its execution [32,33,34,35,36,37,38,39]. To be able to include extensive information from the literature in this scoping review, we used the WHO definition, with it being the most comprehensive one. It encompasses four relevant elements (a–d): “Telemedicine is the delivery of health care services (a), where distance is a critical factor (b), by all health care professionals using information and communication technologies (c) for the exchange of valid information for diagnosis, treatment and prevention of disease and injuries, research and evaluation, and for the continuing education of health care providers, d) all in the interests of advancing the health of individuals and their communities.” [33]. This signifies that telemedicine goes beyond remote health care.

Numerous studies demonstrate that the use of telemedicine in the care of older adults by various medical disciplines and in clinical trials is feasible and acceptable. Stakeholders are satisfied and health outcomes improve, e.g., service utilization and mortality rates decrease. Telemedicine also appears to be cost-effective, saving travel time and eliminating the need for the patient to adjust to an unfamiliar environment [40,41,42,43,44,45,46,47,48,49]. The credibility of evaluation studies, however, appears to be questionable, as there are only a small number of randomized controlled trials (RCT) on the topic [41,46,47,49].

For the NH setting, telemedicine is expected to provide appropriate levels of care to NHRs and their family members and achieve the early detection of a deterioration in health status, thus improving health care in the NH setting [28,50,51]. Telemedicine and telehealth are increasingly applied in NHs, in part because of the COVID-19 pandemic [35,52,53]. Prior to the pandemic, telemedicine was rarely used in inpatient long-term care settings [54]. The old age and multi-morbidity of NHRs could affect the operation of the technical devices. The infrastructure of long-term care facilities was often unsuitable for the use of telemedicine [55]. However, despite these difficulties, telemedicine could still achieve better cooperation and communication between staff from the NH setting and consultation partners (e.g., general physicians, medical specialists, pharmacists, therapists and the staff of emergency/hospital departments). In addition, any ambiguities that NH staff might have about their patients’ care needs, and eventually a decision on their hospitalization, could be clarified or resolved in a direct exchange between health care professionals using telemedicine. Telemedicine offers the chance for more expedient on-site consultation and treatment of the NHR than otherwise might occur, especially in the case of after-hours care. This would increase the chance of preventing a further deterioration of the NHR’s health status and decrease insecurities regarding hospitalization decision-making processes. As a result, the hospitalization of NHRs might be reduced.

The enabling factors, which facilitate the adoption of information and communication technology (ICT) in clinical settings, are the usefulness and ease of use of these services as perceived by medical staff. The direct involvement of stakeholders, a reimbursement for their efforts and easy access play an important role in the successful implementation of telemedicine in NH care [56]. Problems with regards to design, technical concerns and the procurement of equipment, familiarity with ICT and time were the most commonly identified limiting factors [43,57]. So called road maps were developed and recommendations were made to successfully implement telemedicine in healthcare [29,55,58].

This overview of the literature shows that the hospitalization of NHRs is potentially inappropriate and that telemedicine could improve NH quality of care, thus raising the probability that the hospitalization of NHRs due to NHSCs is reduced. NHRs are sometimes incapable of operating the technology themselves. Therefore, this scoping review focused on the NH setting, where health care professionals operated the technology. The primary aim of this study was to identify the types of available evidence regarding telemedicine service use in the NH setting and the hospitalization of NHRs, especially regarding the frequency of the hospitalization of NHRs. After the selection of the literature to be included in our review (see Materials and Methods below), we further analyzed these articles to extract information about the cost-effectiveness of, and the health care staff’s satisfaction with, telemedicine in the NH setting. Finally, the usual topics of interest of a scoping review were considered, namely the identification of possible knowledge gaps and an examination of how the research on this topic was carried out (study design, evidence level and the characteristics of the telemedicine service). With these results, we hope to support research and transfer into clinical practice, so scientists, clinicians and policymakers can prioritize the topics and questions of greatest need [59,60].

To address the two objectives of this scoping review, two research questions were formulated. Our primary research question was: does telemedicine use in the NH setting reduce the frequency of the hospitalization of NHRs?

The secondary research question was: In the identified studies for the primary question, are other outcomes mentioned regarding cost-effectiveness and the health professional’s satisfaction?

## 2. Materials and Methods

Of all the different types of systematic literature reviews and synthesis methods [61,62], we chose the scoping review according to Munn et al.’s guidance and indications for systematic reviewers on the choice of available review methods [63,64], as it appeared to be the study design that is best fit to gather information on the declared aims of this study [65]. This scoping review was conducted systematically according to the quality standards for scoping reviews of the Joanna Briggs Institute ([65], Chapter 11) and the Preferred Reporting Items for Systematic Reviews and Meta-Analyses Extension for Scoping Reviews (PRISMA-ScR) [59,60,65,66,67,68]. We pre-defined the objectives, methods and reporting of our review, which were not changed during the reviewing process.

### 2.1. Search Strategy

PubMed (including Medline, PubMed Central and OldMedline), Livivo, EBSCO (all data bases, including CINAHL and Medline) and Excerpta Medica Journal Storage (JSTOR) were searched for relevant articles between 12 July–30 August 2021 and again on 23 February 2022. Anticipating that authors in the literature would use many synonyms regarding our research topic, we used a broad range of search terms and combined them with Boolean operators. The electronic search strategy for PubMed is presented in Table 1. The other databases were searched accordingly.

For nursing home, we used the keywords nursing home/s, nursing facility/ies, long(-)term care (facility/ies), aged care, care home/s, home/s for the aged, institutional care and residential care. For hospitalization, the search terms were hospitalization (in all its spellings) as well as hospital and/or emergency department admission, with many synonyms and spellings, such as hospital/acute care/emergency admission/transfer*/transition/refer*. For telemedicine, the terms telemedicine, telehealth, Tmed, distance/s based treatment, teleconsultation, information communication technology, ICT, health information technology, external clinical support, ICT based intervention, mobile health, mHealth, digital health, virtual care, telemonitoring and telerehabilitation were used.

### 2.2. Inclusion and Exclusion Criteria

Scientific comparative primary research articles in English and German that investigated telemedicine in a NH setting, in terms of hospitalization and related outcomes (emergency department visits and mortality), or efficiency parameters (time and cost savings) and the satisfaction of medical/nursing staff were included. Included studies concerned quantitative and qualitative comparative studies, e.g., (non-)randomized clinical trials or cohort, case-control, cross-sectional, pre-post and mixed-method studies. If the comparison was to research results by someone else (not working on the same project) or the scientific literature in general, studies were excluded. The methods and setting of data gathering might be too diverse and thus contain bias potential. Research in inpatient care settings similar to NHs, such as rehabilitation centers, long-term care facilities and skilled nursing facilities, were accepted, if the population and provided care would be expected to be comparable to the NH population and care. To yield a high-quality review, only studies published in peer-reviewed journals with impact factor were included. The country of origin and publication year was not a selection criterion, as religious, cultural or social and timely differences in the care of NHRs would be taken into account in the interpretation.

We narrowed the wide range of possible techniques to audio and/or video conferencing/consultation with or without further health ICT-technology integration, thus enabling direct communication and exchange between stakeholders, allowing for early intervention and the building of partnerships with community diagnostic organizations to expedite treatment, ensuring access to clinical nurses or physicians and other specialists. These are all mentioned to be important factors in the decision-making process with regard to the hospitalization of NHRs [2,27,28]. Therefore, studies on pure health information technology exchange (without direct interaction of physician/nursing staff/NHR) were excluded. Publications that concerned conference abstracts, books/book parts, serial articles, reports, opinions, non-scientific essays, study proposals and reviews were also excluded.

### 2.3. Study Selection

In total, the search yielded 1127 results. A total of 490 articles remained after removing duplicates (n = 417), studies published in non-peer reviewed journals or journals without impact factor (n = 25) as well as non-scientific primary articles, book chapters and series (n = 195). The title and abstract screening on these articles was executed independently by author 1 (M.P.V.-D.) and author 2 (S.B.-J.) and differed in the case of 22 articles (4%). A full text review was subjected on 60 essays (the 22 with different screening results and an additional 38 in case title or abstract were not equivocal enough). A hand search (M.P.V.-D.) and citation tracking of included studies yielded 444 additional results. Of these, five were excluded directly (duplicates (n = 3) and non-scientific primary articles, book chapters or series (n = 2)). After title and abstract screening (M.P.V.-D-), six articles remained for full-text review. Of the 66 full texts, 50 were excluded for seven different reasons. Table S1 (Supplement) shows these sources [19,42,69,70,71,72,73,74,75,76,77,78,79,80,81,82,83,84,85,86,87,88,89,90,91,92,93,94,95,96,97,98,99,100,101,102,103,104,105,106,107,108,109,110,111,112,113,114,115,116] with their main reason for exclusion: comparison data/group inept (n = 15); not a NH setting (n = 13); telemedicine just recommended or without audio-/videoconferencing (n = 10); not regarding the hospitalization of NHRs (n = 6); congress abstract (n = 3); not a scientific research paper (n = 2); different author, same study (n = 1).

**Figure 1:**
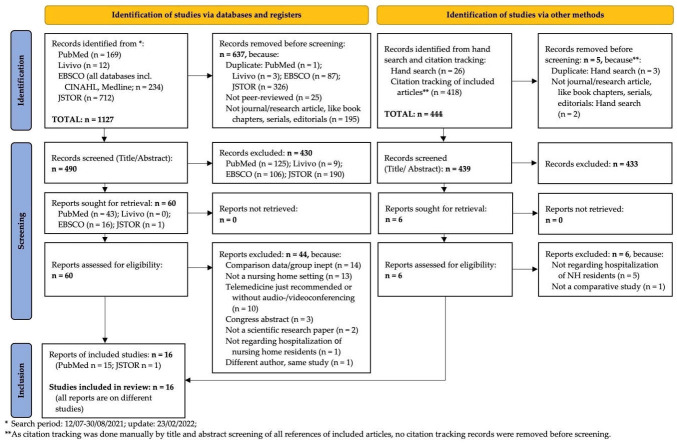
PRISMA flow diagram [67].

A final sample of 16 articles met the in- and exclusion criteria and were selected for duplicate data extraction and analysis (M.P.V.-D.). A Preferred Reporting Items for Systematic Reviews and Meta-Analyses (PRISMA) diagram [67] is shown in Figure 1. The software used for the management of the results of the search were Clarivate Endnote TM 20.2.1 (Philadelphia, PA, USA) and Microsoft Excel 2016 (Redmond, WA, USA).

### 2.4. Data Evaluation

The studies were judged on their level of evidence according to their study design. A fair comparison of the quality of the studies according to Du Prel [117] could not be made, because their study designs, objectives/aims and methodologies used were too different.

### 2.5. Data Extraction

A charting table to extract the data of the included sources was developed in Microsoft Excel 2016 and tested on three articles. After evaluation by the authors, the topics of interest were extended to type of NH, service hours of telemedicine and the purpose of telemedicine. This matrix was based on the recommendations of Peters et al. ([65], Chapter 11) and Newbould et al. [118] and used for duplicate data charting by the first author. The final charting table tool is shown in Table S2 (Supplement).

Data were mapped on the following parent categories:

(1) Study descriptors (year and country of origin, study design, study aim/objective and population descriptors, such as age, gender and (co)morbidities).
(2) Focus (telemedicine intervention details/technology(s)/strategies, involved health care professionals as well as duration of intervention and service hours in which telemedicine was offered (weekdays/after hours) and the purpose of telemedicine, which is operationalized into monitoring, diagnostic and therapeutic properties).
(3) Health outcomes for the primary research question (in this study, emergency department visits, hospitalization and mortality rate).
(4) Procedural/facility outcomes for the secondary research question (in this study, cost-effectiveness and satisfaction of health care professionals).

## 3. Results

Of the final 16 studies that met the criteria for consideration in this scoping review, this study considered the following aspects: year of publication, study design, setting, population, medical aspects such as (co)morbidity and involved medical specialties, characteristics of the telemedicine intervention, evidence on both research questions regarding hospitalization, cost-effectiveness, health care staff’s satisfaction with the telemedicine service and some remaining aspects of interest such as the validity of study results.

**Figure 2:**
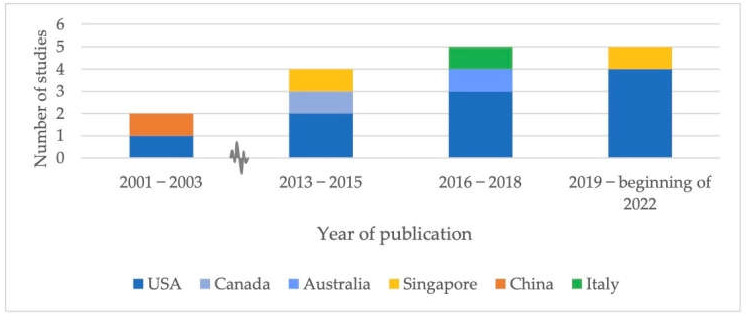
Numbers of included studies by year and country of origin.

The included studies were published between 2001 and 2022, with a publication gap from 2002–2013. Of the studies published in this gap (155), half of them were excluded because they did not concern scientific nor comparative study articles (77/155) and 42% (65/155) because they did not take place in the NH setting. Of the included studies, 10/16 were published after 2016 (Figure 2) [119,120,121,122,123,124,125,126,127,128]. 

A descriptive summary of study and intervention descriptors, with their relevance to this scoping review’s objective, is shown in Table S3 (Supplement). The results of the data extraction are shown in Table S4 (Supplement) and of the data charting in Table 2.

### 3.1. Study Design

The sixteen studies in this scoping review included seven studies with higher credibility: one RCT [121]; five cohort studies (two with a stepped-wedge design [124,129], one retrospective [123], one pilot study [130], one blinded and randomized with integrated pre-post intervention comparison and matching of NHs [131]); and one study with a difference in difference pre-post comparative design and propensity score matching of NHRs [125]. The remaining nine had a lower level of evidence: eight pre-post comparative studies [119,120,122,126,127,132,133,134], of which two were pilot studies [122,132]; and one quality improvement pilot study [128].

### 3.2. Setting

Most of the studies (10/16) were carried out in the USA [119,120,122,123,124,125,128,130,131,133]. A quarter of the projects were carried out in rural areas [120,122,125,127]. Seven studies studied telemedicine use in the nursing home [121,126,127,128,131,132,134] and three in a long-term care (LTC) setting [122,129,130]. Of the other studies, four were in skilled nursing facilities (SNF) [119,120,123,133] and two in both LTC and SNF settings [124,125].

### 3.3. Population

Four studies did not publish data on the exact sample size [122,132,133,134]. These and another three studies [119,127,131] refrained from publishing data on the average age and gender sample size proportion. Partly deducted from the number of beds in a NH or other information, the sample size ranged from 41 to 34,228: 9 of 16 studies had less than 200 NHRs [120,121,127,128,129,130,132,133,134], three ranged between 200–1000 [119,126,131] and the remaining four studies had over a thousand NHRs [122,123,124,125]. Weighted for sample size, the average age was 78 years old (range 75–85 y.) and 63% were female (range 59%–86%), with the oldest population having the highest female share.

### 3.4. (Co)Morbidity and Involved Medical Specialties

Six studies provided detailed [122,124,125,126,129,130] and two superficial information [123,134] on (co)morbidity. Most studies mentioned conditions characteristic of the NH setting, such as neurological (e.g., depression, dementia), respiratory (e.g., COPD, pneumonia), cardiovascular (e.g., infarction, hypertension, CVA), muscular skeletal (e.g., fractures, osteoporosis), dermatological (e.g., pressure ulcers) and urinary disorders. Another five studies provided either information on involved medical specialties [119,132] or no information on this aspect [121,131,133]. The remaining three studies concentrated on one diagnosis (heart failure [120], oral health problems [127] or one subpopulation (NHRs in palliative care [128])).

### 3.5. Telemedicine Intervention

In most studies, the purpose of telemedicine was rarely explicitly mentioned, but three categories could be derived from its description. There were ten single purpose studies: five on diagnostics [119,122,123,127,131], two on therapy [128,130] and three on monitoring [120,121,124]. Of the remaining six with a combined purpose [125,126,129,132,133,134], one study combined all three purposes [125].

Ten of the sixteen studies used additional diagnostic technology in telemedicine care, e.g., a digital stethoscope, vital signs sensory techniques and digitally transmitted photography material [119,120,121,122,123,125,127,129,132,133]. Nine studies included (electronic) medical health record data [119,120,121,123,124,125,129,130,133].

Six projects studied telemedicine on weekdays [121,124,127,129,132,133]. Telemedicine was offered after hours in two projects [119,131], whereas seven studies offered telemedicine around-the-clock (24/7) [120,122,123,125,128,130,134]. In twelve study projects, health professionals had training before using the telemedicine service [119,120,121,122,125,126,127,128,129,130,131,133].

In seven studies, an intermediate telemedicine service was used to establish contact between communicating parties [120,123,126,127,129,130,132,133,134]. Of these, four were integrated in a specific health care model/program, e.g., Supportive Care Program [128], Avera eLTC program [122,125] or Sicilian Tele-Health-Care model [121]. One study used the ECHO-AGE model, without using an intermediate telemedicine service [130]. Of the five studies using a specific health care model, three involved specialists in telemedicine care [121,122,130]. The other six studies with specialists in telemedicine care did not use an intermediate service [120,126,127,129,132,134].

Higher educated nursing staff were involved in 11 of the 16 studies: on the telemedicine consultant site in 5 projects [122,125,128,131,132], in the NH in 4 studies [119,120,123,126] and in 2 studies on both sides of the virtual connection [129,133]. In communicating with physicians and specialists, six studies involved specialized nursing staff [119,120,123,126,129,133]. Administrative staff were part of the external telemedicine service in four studies [121,122,125,131] and deployed in two further projects [120,133]. Four studies involved therapists in the telemedicine health care, all of them in studies with direct telemedicine consultation with the NH [127,129,132,133].

In terms of training [125,126,130,131], a better understanding of what telemedicine is capable of was seen as effective in promoting staff engagement. Engagement [129,131], experience and/or training [125,126], acceptance and eventually direct contact between stakeholders [124] appeared to foster a reduction in hospitalization by telemedicine consultation in NH care. The explanation for reduced hospitalization rates and emergency department visits in studies with higher educated staff on-site in the NH [122,126,127,130], was sought in nursing staff acting as mini-specialists, who either resolve care problems independently or, because of better diagnostic capabilities, make (sufficiently) early detection more likely. Education might also cause a “ripple effect” throughout participating facilities: Other residents, with comparable conditions and symptoms, could benefit from the improved care management [130]. A tabular description of the characteristics of the telemedicine service in the included articles is shown in Table 3.

### 3.6. Outcomes on the Primary Review Question

Twelve of the sixteen studies found a reduction in emergency department visits (ED) and/or the NHR hospitalization rate after the introduction of the use of telemedicine in the nursing home/s [119,120,121,122,123,126,127,128,130,132,133,134], one of which also reported a decrease in mortality [130]. Four studies, all with a higher level of evidence, did not show such an effect [124,125,129,131], though two of them did find a reduction in the hospitalization rate for subgroups. In these studies, NHRs of more engaged facilities [131] or newly admitted NHRs, when telemedicine had already been introduced for a longer period [125], did have lower hospitalization rates. A study in which the consultation-receiving physician showed a moderate acceptance rate of telemedicine advice [124] resulted in no discernible effect. Thus, in fourteen studies, a reduction in the hospitalization rate for NHRs or subgroups of NHRs was found after the introduction of telemedicine. In two studies, ED visits/the hospitalization rate for NHRs did not change significantly [124,129], one of which involved a stakeholder with only a moderate level of acceptance with regards to the telemedicine consultation.

The study results were based on very different methodological approaches in terms of study designs and databases, making a direct comparison of the hospitalization rates of limited value. For instance, three of the four studies that found no or only a partial reduction in hospitalization rate involved an intermediate telemedicine service [124,125,131], with all three not involving specialists. Furthermore, a comparison of the results was complicated by the use of different numerical units for hospitalization rate reductions. One of the two “no-effect” studies [129] and ten of fourteen studies with reductions in hospitalization/ED visits engaged specialized nursing staff [119,120,122,123,125,126,128,131,132,133]. The two “no-effect” studies [124,129] and four of the fourteen studies that (partly) found a reduction in hospitalization rates [121,127,132,133] offered telemedicine services on weekdays. Of the rest of the studies showing lower hospitalization rates, seven offered telemedicine 24/7 [120,122,123,125,128,130,134] and two offered it after hours [119,131]. Nursing homes consulted specialists by telemedicine in eight of the fourteen studies that did find a reduction [120,121,122,126,127,130,132,134] and in one [129] of the two no-effect-studies. Also, nine of fourteen studies that did find a reduction in hospitalization for the study population or subgroups added medical/monitoring diagnostic technology to its telemedicine service [119,120,121,122,123,125,127,132,133], and seven of them used (electronic) health records [119,120,121,123,125,130,133]. These findings are summarized in Table 4.

### 3.7. Outcomes on the Secondary Review Questions

Eleven studies examined cost-effectiveness, three of which were equivocal about whether telemedicine was beneficial from an economic perspective. Four of the eleven study designs were of higher credibility [123,125,129,131], one of which was inconclusive for the economic consideration [125] (Table 5). The handling of assumptions, the issues to be considered, the definition of numeric units (expenditures per NH or NHR, per year/month/study duration, in absolute or relative terms) varied widely across studies. One study mentioned cost savings per NHR per year [125], which appears to be the most appropriate numerical unit, as the number of NHRs per facility varies greatly. This study found cost-savings of USD 5 per long-term care resident per year and could not find any cost-savings in the skilled nursing facility setting. Other studies presented numbers on cost-savings ranging between USD 30,510/NH/year [132] and up to USD 120,000/NH/year in the case of more engaged facilities [131]. In some articles, specifications to indicate expenditure savings were presented [123,126], but they were hard to interpret into practice. Often the indicators used in calculating the cost savings of avoided hospitalizations and emergency department visits were not specified, and further impacts and associated costs were not mentioned explicitly in the included articles [120,131]. If indicators for cost savings were mentioned, they were often interconnected and part of a very complex system on more than one level, as one study showed [132], an avoided ED visit or hospitalization avoids consultation costs, reduces travel time and avoids the transportation costs of the van and staff, emergency department and/or ambulance use, some of which leads to less expense and a higher turnover/clinic caseload of patients by physicians (up to 44%), eventually leading to higher reimbursements of delivered care. Another study enumerated the costs for several items/areas, but provided no conclusive comparison [127]. One study mentioned a relationship between a higher rate of staff training per month and a decline in emergency department costs [126]. 

There were six studies which looked into the satisfaction of staff members. All were positive in this respect [124,125,127,129,132,133]. One study mentioned the acceptance rate of the physician (66%), but this was a specific study on pharmacological recommendations via telemedicine coming from a pharmacist [124]. Another study additionally reported a higher work load felt by 50% of nursing staff after the introduction of telemedicine [132]. Li et al. [125] made positive statements on nursing staff satisfaction with telemedicine by referring to another of their publications on the same project. Lyketsos et al. considered satisfaction on a higher level, speaking of fine-tuning and understanding between facility and hospital staff, i.e., different teams becoming part of a whole [133].

The included studies showed great variation in terms of methods, types of intervention and sample sizes. Sometimes issues with internal validity seem indicated (mentioning deviating hospitalization rates in subsections of the same article [130]; probable 50% overestimation of hospitalization rate reduction by neglecting bed occupancy rates [119]; selection bias by excluding cognitively impaired residents from the telemedicine but not from the usual care group [124]; nullifying intervention impact by measurements to increase internal validity, in that the initial observation served as each person’s own control; hospitalization risk and severity of illness increase with age, whereas telemedicine in general could reduce hospitalization risk [125]; a n observation period that was likely too short for showing intervention impact because of a pilot study design [122,128,130,132]). Furthermore, whether hospitalization or ED visits occurred shortly after the telemedicine consultation was rarely checked [119].

## 4. Discussion

With regard to the status quo of the literature, this scoping review identified 16 comparative studies on telemedicine use in NHs and the effect on hospitalization, and if the studies provided such information, also on cost-effectiveness and health care staff satisfaction with the telemedicine service. A decade after Brignell et al. [47] and Ediripullige et al. [41], this scoping review showed a lack of high quality research on the topic, as only one study (RCT) had a high level of credibility. A total of 14 studies showed positive findings on reductions in hospital and ED visits. These findings are in line with the literature [42,45,49,69,111,135]. As the calculations for time and cost effectiveness in the included studies relied on highly diversified assumptions and decisions on inclusion of mostly interconnected indicators and used different numeric units, elicited cost savings attributed to telemedicine care ranged from marginally to very high. These methodological differences make a comparison with the literature impossible [136]. Therefore, economic calculations were not considered in more detail in this scoping review. The presented satisfaction among health care professionals with telemedicine use in NHs is supported by the views of physicians, residents and families in the literature [49]. The low number of articles regarding satisfaction can be explained by the quantitative nature of the study designs in the included comparative studies, whereas satisfaction is mostly studied by means of qualitative studies.

The publication gap from 2002–2013 can be explained by the novelty of telemedicine in the health care sector: In the introductory period with regard to telemedicine, the focus was on feasibility, stakeholder satisfaction and effectiveness in outpatient and clinical settings rather than on the more specific NH setting. Therefore, and because a hand search and citation tracking of included articles did not lead to the inclusion of new articles, it can be assumed that our search strategy satisfied the purpose of this scoping review. Supported by the literature was also the higher proportion of females with the rising average age of the study population [2,128], the mentioned (co)morbidities belonging to NHSCs [2] and a mostly urban setting [137]. Most studies were conducted in the USA, which might be explained by the USA often being ahead with (research on) innovative health care. In addition, the recent levying of penalties according to the Hospital Readmissions Reduction Program (HRRP) in the USA is likely behind a higher level of interest in studies on measures to keep (re)hospitalization rates low(er) [138,139].

Understanding the contexts, mechanisms and reasons why telemedicine in NHs might be successful, and what outcome measures can be used, is important [118]. The preparation, training, education and involvement of NH staff might increase the engagement of stakeholders. Technical infrastructure and the establishment of new clinical procedures in the implementation process and beyond are prerequisite for the success of telemedicine [111]. The results in our study confirm these influencing factors [124,125,126,129,130,131], even indicating a “dose-effect relationship” [125,131]. Education should also focus on when to involve specialist/physician care by nurses and raise awareness of geriatric care issues [140]. Our findings appear to establish a link between the use of telemedicine in health care and the educational level of nursing staff (nurses acting as mini-specialists after the introduction of telemedicine, or transferring what they learned in one case to the next case causing a ripple effect) [122,126,127,130].

The small number of included studies mainly focused on the effect of telemedicine. Hardly any studies distinguished between the respective effect of audio/video conference/consultation and other telemedicine intervention aspects, such as the addition of medical technology (vital signs sensors, digital stethoscopes, mobile X-ray, etc.), the duty hours of telemedicine services (24/7 or otherwise), the different education levels of stakeholders and the type of telemedicine consultation (video/audio/or a combination) on the hospitalization of NHRs. The increased presence of physicians in NHs during daytime care reduced the hospitalization of NHRs [141]. This effect also appears to apply to physician support via telemedicine. Raising physicians’ awareness of telemedicine, repeated staff training and improving the relationship regarding (mis)trust, (mis)understandings and (mis)perceptions of skill and ability between general practitioners and NH staff would positively influence responses to medical needs and thus the impact of telemedicine [111,142], especially in the case of visual support (videoconferencing) [142]. Telemedicine by audio/video, compared with telemedicine by audio alone, appears to have no advantage in general in the NH setting [89], though videoconferencing appeared to be more effective than telephone consultancy in decision making for acute strokes [44].

Other aspects of interest, e.g., financial and legislative aspects, need further investigation. Telemedicine might resolve liability problems that might arise when NH staff feel insecure in their response to the demanding needs of deteriorating cases. Furthermore, advice to hospitalize was often followed out of fear for liability issues [143]. Research in rural areas regarding telemedicine in the NH setting is still underserved. For areas in which specialist care is not available, so-called hospital-at-home or virtual hospital models were developed with promising outcomes in service use and cost-effectiveness [79,144,145,146,147,148].

### 4.1. Limitations

The limitation of scoping reviews can lie in the limitations of the included studies and in the scoping review itself. Our results should be interpreted with care because the included studies showed huge variation in terms of methodology and different types of study design as well as telemedicine. Cost calculations seldom specified the used indicators [120,131]. The internal validity could be compromised in some studies [119,122,124,125,128,130,132]. Regarding type of long-term inpatient care, residents of SNFs have on average higher health risks, a higher need for care and expenditures, a higher bed-turnover rate because of a shorter average stay than NH/LTC residents [120,125]. These differences might influence the impact of telemedicine care.

Due to its specific question, this scoping review was limited to 16 comparative studies. Furthermore, half of the included studies used small sample sizes, two third were performed in the USA and three quarters in an urban environment, thus limiting the transferability of results. The health care system of the USA, with its financial reimbursement by the Center for Medicare and Medicaid Services (CMS) and the HRRP, is not comparable to European systems. As we did not search for grey literature, the study results might be marred by publication as well as language bias, as there is the tendency in science to publish significant “positive” results in international, English-language journals.

Practical implications concerning (Cyber)security and data protection measures regarding the exchange of medical health information on NHRs by using telemedicine was not explicitly mentioned in the included articles. For future research and especially in implementing telemedicine health care services, special attention should be given to data security and protection aspects.

### 4.2. Recommendations for Future Research

There is need for higher evidence level research. The expectations for telemedicine are high, with it having the potential to greatly impact the future of medicine and profoundly alter the medical landscape [149,150], but these promising expectations still have to be verified. More RCTs could fill this gap in high quality research, as RCTs are still regarded as the study design with the highest robustness in terms of findings [65]. High evidence research is also needed on the effect and underlying mechanisms of facilitators of and barriers to telemedicine in the NH setting. Summarizing the complex and interdepending indicators for cost calculation could lead to more transparency and make the outcomes more comparable. Reporting guide lines (CONsolidated Standards Of Reporting Trials (CONSORT) and its extensions [151,152]) should be followed to raise the possibility of direct comparisons of the results of complex intervention studies, which is of interest for meta-analyses. Regarding barriers and facilitators, underrepresented research subjects of interest connected to telemedicine in the NH setting are integrated diagnostic and monitoring health technology (e. g., vital signs collected via sensors), (electronic) health records as well as telemedicine service hours. General parameters of patient safety and quality of care, such as the engagement/commitment and expert knowledge of health care professionals, should be explored more thoroughly in the context of telemedicine. Attention should also be paid to the form of inpatient elderly care, as long-term care, skilled nursing facilities and NHs appear to be quite different.

### 4.3. Implications for Practice

Apart from acquiring geriatric knowledge and diagnostic skills to reduce NH-sensitive hospitalizations, NHs should consider offering telemedicine all day all week (24/7) and preparing, training, educating and engaging their staff to use telemedicine and potentially using additional electronic medical technology. This would raise the probability of an early detection of a deterioration of the NHR’s health status and could ease the decision making process on when to consult experts by telemedicine to prevent hospitalization and to urge needs-based and NHR-centered health care.

## 5. Conclusions

This scoping review supports the positive impact of telemedicine on reducing emergency department and hospital visits among NHRs, health care cost savings and satisfaction with the telemedicine care provided by health care professionals. As there are few studies regarding telemedicine and hospitalization in the NH setting, one of which is a robust RCT, the level of evidence is still low. Moreover, the studies differed in terms of setting, methodology and publication content, so the results can hardly be compared to one another. Before implementing a telemedicine service, it is important that the staff and users of the service, particularly those in the NH setting, are given adequate education and training. Using guidelines on protocol and reporting can lead to more reproducible and generalizable scientific evidence on the subject.

## Figures and Tables

**Table 1:** Electronic search strategy for PubMed.

Database	PubMed: 4 Search Strings Combined with Boolean Operator
For nursing home	(((((nursing home[MeSH Terms] OR “nursing home”[Title/Abstract] OR “nursing homes”[Title/Abstract] OR “nursing facility”[Title/Abstract] OR “nursing facilities”[Title/Abstract] OR “long-term care”[Title/Abstract] OR “long-term care facility”[Title/Abstract] OR “long-term care facilities”[Title/Abstract] OR “aged care”[Title/Abstract] OR “care home”[Title/Abstract] OR “care homes”[Title/Abstract] OR “home for the aged”[Title/Abstract] OR “homes for the aged”[Title/Abstract] OR “institutional care”[Title/Abstract] OR “residential care”[Title/Abstract]))) AND
For telemedicine	(((((((((((((((((((telemedicine[MeSH Terms]) OR (telemedicine[Title/Abstract])) OR (telehealth[Title/Abstract])) OR (Telemedicine[Title/Abstract])) OR (distance based treatment[Title/Abstract])) OR (teleconsultation[Title/Abstract])) OR (information communication technology[Title/Abstract])) OR (ICT[Title/Abstract])) OR (health information technology[Title/Abstract])) OR (external clinical support[Title/Abstract])) OR (ICT-based intervention[Title/Abstract]))) OR (mobile health[Title/Abstract])) OR (mHealth[Title/Abstract])) OR (digital health[Title/Abstract])) OR (virtual care[Title/Abstract])) OR (telemonitor*[Title/Abstract])) OR (telerehabilitation[Title/Abstract]))))) AND
For hospitalization	((((hospitalization[MeSH Terms]) OR (hospitaliz*[Title/Abstract] OR hospitalis*[Title/Abstract])) OR (((hospital[Title/Abstract] OR hospitals[Title/Abstract])) AND (admit*[Title/Abstract] OR admis*[Title/Abstract] OR transfer*[Title/Abstract] OR refer*[Title/Abstract] OR transition[Title/Abstract])) OR ((“acute care”[Title/Abstract]) AND (admit*[Title/Abstract] OR admis*[Title/Abstract] OR transfer*[Title/Abstract] OR refer*[Title/Abstract] OR transition[Title/Abstract])) OR ((emergency[Title/Abstract]) AND (admit*[Title/Abstract] OR admis*[Title/Abstract] OR transfer*[Title/Abstract] OR refer*[Title/Abstract] OR transition[Title/Abstract]))))

**Table 2:** Data charting results of comparative studies on telemedicine use and hospitalization of NHRs: identifying gaps.

	Source	Baxter et al. 2021 [128]	Catic et al. 2014 [130]	Chess et al. 2018 [119]	Dadosky et al. 2018 [120]	De Luca et al. 2016 [121]	Grabowski et al. 2014 [131]	Hofmeyer et al. 2016 [122]	Hui et al. 2001 [132]	Joseph et al. 2020 [123]	Kane-Gill et al. 2021 [124]	Li et al. 2022 [125]	Low et al. 2019 [126]	Lyketsos et al. 2001 [133]	Stern et al. 2014 [129]	Tynan et al. 2018 [127]	Yeow et al. 2015 [134]
**Data Charting Results**																	
**Evidence Level**																	
*Higher*			X			X	X			X	X	X			X		
*Lower*		X		X	X			X	X				X	X		X	X
**Population Density**																	
*High*		X	X	X		X	X		X	X	X		X	X	X		X
*Low*					X			X				X				X	
**Telemedicine Intervention**																	
*Specific Health Care Model*		X	X			X		X				X					
*Intermediate Telemedicine service*		X		X		X	X	X			X	X					
*Direct Contact between* *Communicating Parties*			X		X				X	X			X	X	X	X	X
*Audio*		X	X	X	X	X	X	X	X	X °	X	X	X	X	X	X	X
*Video*		X	X	X	X	X	X	X	X	X °	X	X	X	X	X	X	X
*Medical Diagnostic/* *Monitoring Technology*				X	X	X		X	X	X		X		X	X	X	
*(Electronic) Health Record on NHR*			X	X	X	X				X	X	X		X	X		
*Telemedicine Training/* *Instruction of Caregivers*		X	X	X	X	X	X	X				X	X	X	X	X	
**Involved Health Professionals**																	
*Specialist*			X		X	X		X	X				X		X	X	X
*Physician*		X		X			X			X	X	X		X			
*Specialized Nursing Staff at Consulted Health Care site*		X					X	X	X			X		X	X		
*Specialized Nursing staff at the NH/SNF/LTC*				X	X					X			X	X	X		
*Nursing Staff at the NH/SNF/LTC*		X	X			X	X	X	X		X	X		X		X	X
*Other*					1	1	1	1,2,3	4		3	1,2		1,4	4	4	
**Intervention Properties**																	
*Diagnostic*				X			X	X	X	X		X				X	X
*Therapeutic*		X	X									X	X	X	X		X
*Monitoring*					X	X			X		X	X	X	X	X		
**Review Question 1**																	
*ED Visits*						↓*		↓*	↓	↓		↓	↓		=	↓	↓
*Hospitalization Rate*		↓	↓	↓	↓	↓*	=/↓	↓*	↓	↓	=	=/↓	↓	↓	=		↓
*Mortality Rate*			↓														
**Review Question 2**																	
*Cost-Effectiveness*				↑	↑		↑		↑	↑		=/↑	↑	↑	↑	↑?	↑/↓
*Satisfaction/Well-Being of Health Care Professionals*									↑		↑	↑		↑	↑	↑	
**Duty Hours in Telemedicine**																	
*Weekdays*						X			X		X		n. i.	X	X	X	
*After Hours/Weekends*				X			X						n. i.				
*24/7*		X	X		X			X		X		X	n. i.				X

Abbreviations: ED: Emergency Department; NHR: nursing home resident; X: involved; n. i.: not indicated; ↓: goes down; ↑: goes up; * admission to health care service/hospital transfer, interpreted as ED visit and/or hospitalization; ° telemedicine consultation: interpreted as audio-/video-consultation; ?: inconclusive study results. Other health professionals in telemedicine service: (1) administrative staff (e.g., medical secretary, operator, coordinator), (2) telemedicine hub director, (3) pharmacist, (4) therapist (e.g., physio-/occupational/oral health)

**Table 3:** Study and intervention descriptors of the 16 included studies.

Regarding the 16 Included Studies [119,120,121,122,123,124,125,126,127,128,129,130,131,132,133,134]:		Number of Studies	References
**Study design**	*Higher evidence level ^*	7	[121,123,124,125,129,130,131]
	*Lower evidence level °*	9	[119,120,122,126,127,128,132,133,134]
**Facility**	*Nursing home (NH)*	7	[121,126,127,128,131,132,134]
	*Long-term care facility (LTC)*	3	[122,129,130]
	*Skilled nursing facility (SNF)*	4	[119,120,123,133]
	*LTC and SNF*	2	[124,125]
**Purpose of** **telemedicine**	*Diagnosis*	5	[119,122,123,127,131]
	*Therapy*	2	[128,130]
	*Monitoring*	3	[120,121,124]
	*Combination of purposes*	6	[125,126,129,132,133,134]
**Establishing contact between communicating parties via telemedicine**	*Directly by hospital* *or nursing home itself*	9	[120,123,126,127,129,130,132,133,134]
	*By an intermediate service*	7	[119,121,122,124,125,128,131]
**Consultation by**	*Specialist*	9	[120,121,122,126,127,129,130,132,134]
	*Physician*	7	[119,123,124,125,128,131,133]
**Nursing staff**	*Specialized nursing staff* *at telemedicine consultant site*	5	[122,125,128,131,132]
	*Specialized nursing staff at NH*	4	[119,120,123,126]
	*No specialized nursing staff*	5	[121,124,127,130,134]
	*Specialized staff at both telemedicine consultant site as well as NH*	2	[129,133]
**Consultation** **between**	*Specialist/physician and* *specialized NH nursing staff*	6	[119,120,123,126,129,133]
	*Specialist/physician and* *NH nursing staff*	10	[121,122,124,125,127,128,130,131,132,134]
**Telemedicine** **service offered on ^^**	*Weekdays*	6	[121,124,127,129,132,133]
	*Both weekdays and after hours*	7	[120,122,123,125,128,130,134]
	*After hours*	2	[119,131]
**Use of medical/monitoring diagnostic technology**	*Yes*	10	[119,120,121,122,123,125,127,129,132,133]
	*No*	6	[124,126,128,130,131,134]
**Use of (electronic) health record**	*Yes*	9	[119,120,121,123,124,125,129,130,133]
	*No*	7	[122,126,127,128,131,132,134]

^ Higher evidence level study design: RTC, cohort studies. ° Lower evidence level study design: pre-post comparisons, quality improvement pilot study. ^^ One study did not indicate its service hours [126].

**Table 4:** Reduction in emergency department visits, hospitalization and/or mortality and level of evidence of the study design.

		Reduction in Emergency Department Visits, Hospitalization, and/or Mortality		Total Number of Studies
		Yes	No	
**Level of evidence (credibility) according to study design**	*higher*	**3** [121,123,130]	4 * [124,125,129,131]	**7**
	*lower*	**9** [119,120,122,126,127,128,132,133,134]	**0**	**9**
**Total number of studies**		**12**	4 *	**16**

* Two studies did find a reduction in hospitalization/emergency department rates for sub-groups of NHRs in cases where staff were more engaged or more experienced regarding telemedicine use in NH [125,131].

**Table 5:** Cost savings and level of evidence of the study design.

		Cost Savings		Total Number of Studies
		Yes	Inconclusive/No	
**Level of evidence (credibility) according to study design**	*higher*	**3** [123,129,131]	1 * [125]	**4**
	*lower*	**5** [119,120,126,132,133]	**2** [127,134]	**7**
**Total number of studies**		**8**	**3**	**11**

* This study did find a reduction in hospitalization/emergency department rates for sub-groups of NHRs in case where staff were more experienced regarding telemedicine use in NHs [125].

## Data Availability

All data generated or analyzed during this study are either included in this published article, including Supplementary Materials, or available through the detailed reference list. No original datasets are presented because this is a review of already existing literature.

## Supplementary Materials

The Supplement with supporting information can be downloaded at: https://www.mdpi.com/article/10.3390/ijerph191912944/s1. It contains four tables: Table S1: Excluded studies following full text review: Details on database source and primary reason for exclusion; Table S2: The data extraction instrument; Table S3: Included studies: Summary of study characteristics and population; Table S4. Included studies: Details on intervention and main findings. Reference numbers, mentioned in Tables S1–S4 and in the reference list at the end of the Supplement, refer to the according reference numbers in the text of the main article.

## Abbreviations

APN	advanced practice nurse
C	control group
CMS	Center for Medicare and Medicaid Services
ED	Emergency Department
e.g.,	for instance
etc.	etcetera
HRRP	Hospital Readmissions Reduction Program
I	intervention group
ICT	information and communication technology
JBI	Joanna Briggs Institute;
LTC	long-term care facility/facilities
M.P.V.-D.	author’s initials of first author
n.a.	not applicable
n.i.	not indicated
NH	nursing home/s;
NHR	nursing home resident/s
NHSC	nursing home-sensitive condition/s
ONC	U.S. Department of Health and Human Services’ Office of the National Coordinator for Health Information Technology
PRISMA-ScR	Preferred Reporting Items for Systematic reviews and Meta-Analyses extension for Scoping Reviews.
PU	pressure ulcer
RCT	randomized controlled trial
S.B.-J.	author’s initials of second author
SNF	skilled nursing facility/facilities
Tmed	telemedicine
USD	United States Dollar/s
X (Table 2)	involved
↓ (Table 2)	goes down
↑ (Table 2)	goes up
? (Table 2)	ambivalent study results
