# Supplementary material for: Nursing Home-Sensitive Hospitalizations and the Relevance of Telemedicine: A Scoping Review
Source: Int J Environ Res Public Health. 2022 Oct 10;19(19):12944. doi: 10.3390/ijerph191912944 (PMC9566431; doi:10.3390/ijerph191912944)
Supplement: Supplementary file 1 [file ijerph-19-12944-s001.zip › ijerph-1899753-supplementary.pdf]

Supplementary Materials to Scoping Review (original research):

Nursing Home-Sensitive Hospitalizations and the Relevance of Telemedicine use: A Scoping Review

Authors: Maria Paula Valk-Draad, Sabine Bohnet-Joschko
Institution: Chair of Health Care Management and Innovation, Faculty of Management, Economics, and Society, Witten/Herdecke University, Witten, Germany
Copyright: License: Creative Commons CC-BY Attribution 4.0 International. Credit must be given to the creators

Content

Table S1. Excluded studies following full text review: Details on database source and primary reason for exclusion 2
Table S2. The data extraction instrument 6
Table S3. Included studies: Summary of study characteristics and population 7
Table S4. Included studies: Details on intervention and main findings 11

NOTE:

Reference numbers, mentioned in Tables S1-S4 and in the reference list at the end of the Supplement, refer to the according reference numbers in the text of the main article "Nursing Home-Sensitive Hospitalizations and the Relevance of Telemedicine use: A Scoping Review". Literature references of the 16 included studies are integrated at the end of this document. Other literature references are integrated in the respective table.

Table S1. Excluded studies following full text review: Details on database source and primary reason for exclusion

**No.^**	**Excluded source**	**Database**			**Reason for exclusion***						
		**Pub-Med**	**EBSCO**	**Hand search**	**A**	**B**	**C**	**D**	**E**	**F**	**G**
[69]	Archbald-Pannone, L.; Harris, D.; Steele, R.; Kaur, J.; Albero, K.; Mutter, J.; Cattell-Gordon, D.; Rheuban, K., Virtual Daily Rounding for COVID-19 Facility Outbreaks: A Standardized Telehealth-Centered Approach May Reduce Hospital Transfers and Mortality. *Telemed J E Health* **2021,** *27*, 915-918. https://doi.org/10.1089/tmj.2021.0108.	X			X						
[70]	Chen, Y. H.; Ho, Y. L.; Huang, H. C.; Wu, H. W.; Lee, C. Y.; Hsu, T. P.; Cheng, C. L.; Chen, M. F., Assessment of the clinical outcomes and cost-effectiveness of the management of systolic heart failure in Chinese patients using a home-based interven-tion. *The Journal of international medical research* **2010,** *38*, 242-252. https://doi.org/10.1177/147323001003800129.		X			X					
[71]	Clarke, M.; Bratan, T.; Kulkarni, S.; Jones, R., The impact of remote patient monitoring in managing silent myocardial infarction in a residential home setting. *Anadolu Kardiyol Derg* **2007,** *7 Suppl 1*, 186-188.	X			X						
[72]	Crotty, M.; Killington, M.; van den Berg, M.; Morris, C.; Taylor, A.; Carati, C., Telerehabilitation for older people using off-the-shelf applications: acceptability and feasibility. *J Telemed Telecare* **2014,** *20*, 370-376. https://doi.org/10.1177/1357633x14552382.	X						X			
[73]	Davis, C.; Bender, M.; Smith, T.; Broad, J., Feasibility and Acute Care Utilization Outcomes of a Post-Acute Transitional Tele-monitoring Program for Underserved Chronic Disease Patients. *Telemedicine journal and e-health: the official journal of the American Telemedicine Association* **2015,** *21*, 705-713. https://doi.org/10.1089/tmj.2014.0181.		X			X					
[74]	DeBlois, D.; Millefoglie, M., Telehealth: Enhancing collaboration, improving care coordination. *Nursing management* **2015,** *46*, 10-12. https://doi.org/10.1097/01.NUMA.0000465402.45956.99.		X			X					
[75]	Dy, P.; Morin, P. C.; Weinstock, R. S., Use of telemedicine to improve glycemic management in a skilled nursing facility: a pilot study. *Telemed J E Health* **2013,** *19*, 643-645. https://doi.org/10.1089/tmj.2012.0274.			X				X			
[76]	Eckardt, P.; Guran, R.; Hennemyre, J.; Arikupurathu, R.; Poveda, J.; Miller, N.; Katz, R.; Frum, J., Hospital affiliated long term care facility COVID-19 containment strategy by using prevalence testing and infection control best practices. *American Journal of Infection Control* **2020,** *48*, 1552-1555. https://doi.org/10.1016/j.ajic.2020.06.215.		X		X						
[77]	Errasti, U.; Pérez-Fernández, N.; Elola, M.; Fuertes, A.; Vaquero, M.; Berroeta, A.; Alberdi, A.; Zubeltzu, B.; Mitxelena, A.; Zu-beldia, J. J.; Aranegui, P.; Arteche, J. M., Evolution of a multipronged and multi-intevention strategy for patients with high comorbidity. *International Journal of Integrated Care (IJIC)* **2019,** *19,* 43. https://doi.org/10.5334/ijic.s3043.		X						X		
[78]	Fong, R.; Tsai, K. C. F.; Tong, M. C. F.; Lee, K. Y. S., Management of Dysphagia in Nursing Homes During the COVID-19 Pandemic: Strategies and Experiences. *SN comprehensive clinical medicine* **2020**, *2*, 1361-1365; https://doi.org/10.1007/s42399-020-00464-0.		X				X				
[79]	Freedman, M.; Binns, M. A.; Serediuk, F.; Wolf, M. U.; Danieli, E.; Pugh, B.; Gale, D.; Abdellah, E.; Teleg, E.; Halper, M.; et al. Virtual Behavioural Medicine Program: A Novel Model of Care for Neuropsychiatric Symptoms in Dementia. *J Alzheimer's Dis* **2022,** *86*, 1169-1184. https://doi.org/10.3233/jad-215403.	X				X					
[42]	Friedman, D.M.; Goldberg, J.M.; Molinsky, R.L.; Hanson, M.A.; Castaño, A.; Raza, S.-S.; Janas, N.; Celano, P.; Kapoor, K.; Telaraja, J.; et al. A Virtual Cardiovascular Care Program for Prevention of Heart Failure Readmissions in a Skilled Nursing Facility Population: Retrospective Analysis. *JMIR Cardio* **2021**, *5*, e29101. https://doi.org/10.2196/29101.	X			X						
[80]	Gomes, V. M. G.; Butzke, B.; Kubitschek, M., Developing an innovative, integrated care pathway for PMV patients. *International Journal of Integrated Care (IJIC)* **2016,** *16*, A177. https://doi.org/10.5334/ijic.2725.		X						X		
[81]	Gray, L. C.; Edirippulige, S.; Smith, A. C.; Beattie, E.; Theodoros, D.; Russell, T.; Martin-Khan, M., Telehealth for nursing homes: the utilization of specialist services for residential care. *J Telemed Telecare* **2012,** *18*, 142-146. https://doi.org/10.1258/jtt.2012.SFT105.	X			X						

No.^	Excluded source	Database			Reason for exclusion*						
		Pub-Med	EBSCO	Hand search	A	B	C	D	E	F	G
(Table S1 continued)											
[82]	Guilfoyle, C.; Wootton, R.; Hassall, S.; Offer, J.; Waaren, M.; Smith, D., Preliminary experience of allied health assessments delivered face to face and by videoconference to a residential facility for elderly people. *Journal of Telemedicine and Telecare* **2003,** *9*, 230-233. https://doi.org/10.1258/135763303322225571.			X				X			
[83]	Guilfoyle, C.; Wootton, R.; Hassall, S.; Offer, J.; Warren, M.; Smith, D.; Eddie, M., User satisfaction with allied health services delivered to residential facilities via videoconferencing. *Journal of Telemedicine and Telecare* **2003,** *9(1_suppl)*, 52-54. https://doi.org/10.1258/135763303322196349.			X				X			
[84]	Harris, D. A.; Archbald-Pannone, L.; Kaur, J.; Cattell-Gordon, D.; Rheuban, K. S.; Ombres, R. L.; Albero, K.; Steele, R.; Bell, T. D.; Mutter, J. B., Rapid Telehealth-Centered Response to COVID-19 Outbreaks in Postacute and Long-Term Care Facilities. *Telemed J E Health* **2021,** *27*, 102-106. https://doi.org/10.1089/tmj.2020.0236.	X			X						
[85]	Harry, M.; Woehrle, T.; Renier, C.; Furcht, M.; Enockson, M., Predictive utility of the Activity Measure for Post-Acute Care '6-Clicks' short forms on discharge disposition and effect on readmissions: a retrospective observational cohort study. *BMJ open* **2021,** *11*, e044278. https://doi.org10.1136/bmjopen-2020-044278.		X				X				
[86]	Hassall, S.; Wootton, R.; Guilfoyle, C., The cost of allied health assessments delivered by videoconference to a residential facility for elderly people. *J Telemed Telecare* **2003,** *9*, 234-237. https://doi.org10.1258/135763303322225580.			X		X		X			
[87]	Hernández, C.; Alonso, A.; Garcia-Aymerich, J.; Grimsmo, A.; Vontetsianos, T.; García Cuyàs, F.; Altes, A. G.; Vogiatzis, I.; Garåsen, H.; Pellise, L.; Wienhofen, L.; Cano, I.; Meya, M.; Moharra, M.; Martinez, J. I.; Escarrabill, J.; Roca, J., Integrated care services: lessons learned from the deployment of the NEXES project. Int J Integr Care 2015, 15, e006. https://doi.org/10.5334/ijic.2018.	X									
[88]	Hicks, S. A.; Cimarolli, V. R., The effects of telehealth use for post-acute rehabilitation patient outcomes. J Telemed Telecare 2018, 24, 179-184; https://doi.org/10.1177/1357633x16686771.	X					X				
[89]	Hullick, C.; Conway, J.; Hall, A.; Murdoch, W.; Cole, J.; Hewitt, J.; Oldmeadow, C.; Attia, J., Video-telehealth to support clinical assessment and management of acutely unwell older people in Residential Aged Care: a pre-post intervention study. *BMC Geriatr* **2022,** *22*, 40. https://doi.org/10.1186/s12877-021-02703-y.	X			X						
[90]	Kaldy, J., Telehealth enables senior care pharmacists to reach beyond facility walls. *Consult Pharm* **2005,** *20*, 558-567. https://doi.org/10.4140/tcp.n.2005.558.	X								X	
[91]	Lapane, K. L.; Hughes, C. M.; Daiello, L. A.; Cameron, K. A.; Feinberg, J., Effect of a pharmacist-led multicomponent intervention focusing on the medication monitoring phase to prevent potential adverse drug events in nursing homes. *Journal of the American Geriatrics Society* **2011,** *59*, 1238-1245. https://doi.org/10.1111/j.1532-5415.2011.03418.x.	X					X				
[92]	Lewis, G., Career development. Virtual wards: real nursing. *Primary Health Care* **2007,** *17*, 47. https://doi.org/10.7748/phc.17.6.47.s22.		X							X	
[93]	Mahlknecht, A.; Krisch, L.; Nestler, N.; Bauer, U.; Letz, N.; Zenz, D.; Schuler, J.; Fährmann, L.; Hempel, G.; Flamm, M.; Osterbrink, J., Impact of training and structured medication review on medication appropriateness and patient-related outcomes in nursing homes: results from the interventional study InTherAKT. *BMC Geriatr* **2019,** *19*, 257. https://doi.org/10.1186/s12877-019-1263-3.	X					X				
[94]	Martin-Khan, M. G.; Edwards, H.; Wootton, R.; Counsell, S. R.; Varghese, P.; Lim, W. K.; Darzins, P.; Dakin, L.; Klein, K.; Gray, L. C., Reliability of an Online Geriatric Assessment Procedure Using the interRAI Acute Care Assessment System. *J Am Geriatr Soc* **2017,** 65, 2029-2036. https://doi.org/10.1111/jgs.14895.	X				X					
[95]	Mateos-Nozal, J.; Pérez-Panizo, N.; Zárate-Sáez, C. M.; Vaquero-Pinto, M. N.; Roldán-Plaza, C.; Mejía Ramírez-Arellano, M. V.; Sánchez García, E.; Garza-Martínez, A. J.; Cruz-Jentoft, A. J., Proactive Geriatric Comanagement of Nursing Home Patients by a New Hospital-Based Liaison Geriatric Unit: A New Model for the Future. *J Am Med Dir Assoc* **2022,** *23*, 308-310. https://doi.org/10.1016/j.jamda.2021.12.006.	X			X						
[96]	Mullen-Fortino, M.; Sites, F. D.; Soisson, M.; Galen, J., Innovative use of tele-ICU in long-term acute care hospitals. *AACN advanced critical care* **2012,** *23*, 330-336. https://doi.org/10.1097/NCI.0b013e31825dfeff.		X			X					

No.^	Excluded source	Database			Reason for exclusion*						
		Pub-Med	EBSCO	Hand search	A	B	C	D	E	F	G
(Table S1 continued)											
[97]	Nishita, C. M.; Johnson, J.; Silverman, M.; Ozaki, R.; Koller, L., Hawai'i's "Going Home Plus" project: a new option to support community living. *Hawaii Med J* **2009,** *68*, 166-168.	X				X					
[98]	Ohligs, M.; Stocklassa, S.; Rossaint, R.; Czaplik, M.; Follmann, A., Employment of Telemedicine in Nursing Homes: Clinical Requirement Analysis, System Development and First Test Results. *Clin Interv Aging* **2020,** *15*, 1427-1437. https://doi.org/10.2147/cia.S260098.	X			X						
[99]	Ohta, R.; Ryu, Y., Improvement in palliative care quality in rural nursing homes through information and communication technology-driven interprofessional collaboration. *Rural Remote Health* **2021,** *21*, 6450. https://doi.org/10.22605/rrh6450.	X					X				
[100]	Ošmera, O.; Bulava, A., The benefits of remote monitoring in long-term care for patients with implantable cardioverter-defibrillators. *Neuro Endocrinol Lett* **2014,** *35 Suppl 1*, 40-48.	X				X					
[101]	Pallawala, P. M.; Lun, K. C., EMR-based TeleGeriatric system. *Stud Health Technol Inform* **2001,** *84 (Pt 1)*, 849-853.	X			X						
[102]	Peterson-Sgro, K., Reducing acute care hospitalization and emergent care use through home health disease management: one agency's success story. *Home Healthcare Nurse* **2007,** *25*, 622-627. https://doi.org/10.1097/01.NHH.0000298930.45717.29.		X			X					
[103]	Rantz, M.; Lane, K.; Phillips, L. J.; Despins, L. A.; Galambos, C.; Alexander, G. L.; Koopman, R. J.; Hicks, L.; Skubic, M.; Miller, S. J., Enhanced registered nurse care coordination with sensor technology: Impact on length of stay and cost in aging in place housing. *Nursing outlook* **2015,** *63*, 650-655. https://doi.org/10.1016/j.outlook.2015.08.004.		X				X				
[104]	Rantz, M. J.; Skubic, M.; Alexander, G.; Aud, M. A.; Wakefield, B. J.; Galambos, C.; Koopman, R. J.; Miller, S. J., Improving nurse care coordination with technology. *CIN: Computers, Informatics, Nursing* **2010,** *28*, 325-332. https://doi.org/10.1097/NCN.0b013e3181f69838.		X		X						
[19]	Rantz, M.J.; Popejoy, L.; Vogelsmeier, A.; Galambos, C.; Alexander, G.; Flesner, M.; Crecelius, C.; Ge, B.; Petroski, G. Successfully Reducing Hospitalizations of Nursing Home Residents: Results of the Missouri Quality Initiative. *J. Am. Med. Dir. Assoc.* **2017**, *18*, 960–966. https://doi.org/10.1016/j.jamda.2017.05.027.	X					X				
[105]	Roques, C.; Hovanec, L., Tele-medicine and the reduction of psychiatric admissions for dementia patients -- social work as the core discipline. *Journal of Social Work in Long-Term Care* **2002,** *1*, 35-41. https://doi.org/10.1300/J181v01n01_09.		X								X
[106]	Sabesan, S.; Larkins, S.; Evans, R.; Varma, S.; Andrews, A.; Beuttner, P.; Brennan, S.; Young, M., Telemedicine for rural cancer care in North Queensland: bringing cancer care home. *Aust J Rural Health* **2012,** *20*, 259-264. https://doi.org/10.1111/j.1440-1584.2012.01299.x.	X				X					
[107]	Sävenstedt, S.; Bucht, G.; Norberg, L.; Sandman, P. O., Nurse-doctor interaction in teleconsultations between a hospital and a geriatric nursing home. *J Telemed Telecare* **2002,** *8*, 11-18. https://doi.org/10.1258/1357633021937406.			X	X						
[108]	Specht, J. K.; Wakefield, B.; Flanagan, J., Evaluating the cost of one telehealth application connecting an acute and long-term care setting. *J Gerontol Nurs* **2001,** *27*, 34-39. https://doi.org/10.3928/0098-9134-20010101-11.	X			X						
[109]	Tappen, R. M.; Newman, D.; Huckfeldt, P.; Yang, Z.; Engstrom, G.; Wolf, D. G.; Shutes, J.; Rojido, C.; Ouslander, J. G., Evaluation of Nursing Facility Resident Safety During Implementation of the INTERACT Quality Improvement Program. *Journal of the American Medical Directors Association* **2018,** *19*, 907. https://doi.org/10.1016/j.jamda.2018.06.017.		X				X				
[110]	Vitacca, M.; Paneroni, M.; Grossetti, F.; Ambrosino, N., Is There Any Additional Effect of Tele-Assistance on Long-Term Care Programmes in Hypercapnic COPD Patients? A Retrospective Study. *COPD* **2016,** *13*, 576-582. https://doi.org/10.3109/15412555.2016.1147542.	X				X					
[111]	Wade, V.; Whittaker, F.; Hamlyn, J., An evaluation of the benefits and challenges of video consulting between general practiti-oners and residential aged care facilities. *J Telemed Telecare* **2015,** *21*, 490-493. https://doi.org/10.1177/1357633x15611771.	X			X						

No.^	Excluded source	Database			Reason for exclusion*						
		Pub-Med	EBSCO	Hand search	A	B	C	D	E	F	G
(Table S1 continued)											
[112]	Watanabe, T. K.; Esquenazi, A.; Flanagan, S., The Transformation of the Rehabilitation Paradigm Across the Continuum of Care. *Pm r* **2018,** *10(9 Suppl 2)*, S264-S271. https://doi.org/10.1016/j.pmrj.2018.08.381.	X			X						
[113]	Whitson, H. E.; Hastings, S. N.; Lekan, D. A.; Sloane, R.; White, H. K.; McConnell, E. S., A quality improvement program to enhance after-hours telephone communication between nurses and physicians in a long-term care facility. *J Am Geriatr Soc* **2008,** 56, 1080-1086. https://doi.org/10.1111/j.1532-5415.2008.01714.x.			X				X			
[114]	Wilmink, G.; Dupey, K.; Alkire, S.; Grote, J.; Zobel, G.; Fillit, H. M.; Movva, S., Artificial Intelligence-Powered Digital Health Platform and Wearable Devices Improve Outcomes for Older Adults in Assisted Living Communities: Pilot Intervention Study. *JMIR Aging* **2020,** *3*, e19554. https://doi.org/10.2196/19554.	X					X				
[115]	Yoon, J.; Chang, E.; Rubenstein, L. V.; Park, A.; Zulman, D. M.; Stockdale, S.; Ong, M. K.; Atkins, D.; Schectman, G.; Asch, S. M., Impact of Primary Care Intensive Management on High-Risk Veterans' Costs and Utilization A Randomized Quality Improvement Trial. *Annals of internal medicine* **2018,** *168*, 846-854. https://doi.org/10.7326/M17-3039.	X				X					
[116]	Zubeltzu, B.; Mitxelena, A.; Alberdi, A.; Elola, M.; Errasti, U.; Berroeta, A.; Vaquero, M.; Fuertes, A.; Perez, N.; Echegaray, M.; Huertas, I.; Emparanza, J. I.; Basabe, I.; Belastegi, A.; Goicoechea, X.; Agirre, C.; Alvarez, M., Multipronged management stra-tegy for patients with complex needs using an integrated organizational model. *International Journal of Integrated Care (IJIC)* **2018,** *18(S2)*, 14. https://doi.org/10.5334/ijic.s2014.		X						X		
	**Total number of studies**	**28**	**16**	**6**	**15**	**13**	**10**	**6**	**3**	**2**	**1**

* *A: Comparison data/group inept: 15*
B: Not a Nursing Home (NH) setting: 13
C: Telemedicine just recommended or without audio-/ videoconferencing: 10
D: Not regarding hospitalization of NH residents: 6
E: Congress Abstract: 3
F: Not a scientific research paper: 2
G: Different author, same study: 1

^ *Reference numbers, mentioned in this table refer to the according reference numbers in the text of the main article "Nursing Home-Sensitive Hospitalizations and the Relevance of Telemedicine use: A Scoping Review"*

Table S2. The data extraction instrument

Data extraction with an Excel table with the following content, specified in rows and columns. Extracted data for each source is displayed in Supplementary Table 3 (in this supplementary file) and Table

**Source**	**Year of publication**	**Country and region of origin**	**Journal abbreviation**	**Impact factor**	**Aim/ objective of study**	**Urban/ rural study area**	**Study design**	**Level of evidence of study design**	**Population**					
									**NH/ SNF/ LTC**	**NHR**	**Mean age (years old)**	**Gender**		**(Co)Morbidity Reason for telemedicine presentation of NHR**
												**m**	**f**	
Author *et al.* Year (Reference number)														

Abbreviations: NH: nursing home; LTC: long-term care facility; SNF: skilled nursing facility; NHR: nursing home resident; n.i: not indicated; I: Intervention group; C: Control group

(Table S2 continued)

**Source**	**Intervention**	**Facility**			**Duration**	**Service on**		**Evidence for the review's primary question**	**Evidence for the review's secondary question**	**Purpose of the study (diagnosis, therapy, monitoring)**
		**NH**	**SNF**	**LTC**		**Weekdays**	**After hours**			
Author *et al.* Year (Reference number)										

Abbreviations: NH: nursing home; NHR: nursing home resident; SNF: skilled nursing facility; LTC: long-term care facility; ED: Emergency Department; APN: Advanced Practice Nurse; Tmed: telemedicine; I:intervention group; C: control group; PU: Pressure Ulcer; n.i.: not indicated; n.a.: not applicable

Table S3. Included studies: Summary of study characteristics and population

Source	Year of publication	Country of origin	Journal abbreviation	Impact factor	Aim/ objective of study	Study design	Population					
							NH/ SNF/ LTC	NHR	Mean age (years old)	Gender		(Co)Morbidity<br>Reason for telemedicine presentation of NHR
										m	f	
Baxter et al. 2021 [128]	2021	USA	J Hosp Palliat Nurs.	0.9	The outcomes of the pilot on provision of palliative care specialists by **telehealth** in **nursing homes** in real time were to measure changes in code status, Medical Order for Life-Sustaining Treatment (MOLST) form completion, and **rehospitalizations**.	quality improvement pilot project	8	(I): 21 (51% of 41 eligible NHR)<br>(C): 20	48%>85 yrs	14%	86%	Palliative care with seriously ill patients. Reason for consult: 86% goals-of-care discussions, 14% symptom management.
Catic et al. 2014 [130]	2014	USA	J Am Med Dir Assoc.	4.4	To design, implement, and assess the pilot phase of an innovative, remote case-based **videoconsultation program** called ECHO-AGE to improve the quality of clinical care and to establish a learning community of primary care providers and specialists. ECHO-AGE links experts in the management of behavior disorders in patients with dementia to **nursing home care** providers. In this pilot compliant with non-compliant residents were compared **on resident improvement, hospitalization and mortality rate**.	Pilot cohort study	11	47	82 (±12.6)	12 (26%)	35 (74%)	Dementia (83%) and/or delirium (36%) related behavioural issues; CVA (23%); depression (68%); hypertension (83%); Parkinson's (6%); chronic renal failure (15%); other psychiatric disorder (28%); initial average number of medication 14 (68% of NHR on antidepressants, 44% on antipsychotics);<br>Reason for consult: 89%: agitation, intrusiveness, paranoia, increase in confusion.
Chess et al. 2018 [119]	2018	USA	Am J Manag Care.	1.7	To evaluate the effectiveness of an after-hours **telemedicine**-enabled bedside clinical evaluation prior to unscheduled **emergency department transfers** from **skilled nursing facilities.**	pre-post intervention comparison	1	313	n.i.	n.i.	n.i.	Involved specialties: 15% gastrointestinal (bleed, vomitting), 15% fall, 9% respiratory (pneumonia), 8% neurology (seizure), 7% genitourinary (UTI), 7% fever, 7% skin, 6% cardiology (chest pain, hypotension), 5% musculo-skeletal (pain), 5% catheter (dislodged), 4% endocrine (hypoglycemia), 4% laboratory, 6% other
Dadosky et al. 2018 [120]	2018	USA	Telemed J E Health.	1.9	To investigate whether **telemanagement** of heart failure patients throughout the post-acute continuum of care would reduce **rehospitalization rates** and improve patient self-care knowledge and satisfaction, and if incorporating the use of point-of-care testing within the **skilled nursing facility** would allow for quicker medical intervention.	pre-post intervention comparison	1	(I): 49 (15%% of 323 eligible NHR)<br>(C): 92, historical control group	81	18 (37%)	31 (63%)	primary or secondary diagnosis of heart failure NYHA class IV
De Luca et al. 2016 [121]	2016	Italy	Aging Clin Exp Res.	2.7	The purpose of this study was to develop a novel Sicilian **Tele-Health-Care** model for elderly living in a **nursing home** and to evaluate its effectiveness, by ensuring the highest level of assistance continuity with a multi-parametric vital sign monitoring, and periodic neurological and psychological tele-consulting, on health outcomes, and **admission to health services** (e.g., hospitalization).	RCT	1	59<br>(I): 32<br>(C): 27	79 (±9.2)	19 (32%)<br>11 (I)<br>8 (C)	40 (68%)<br>21 (I)<br>19 (C)	n.i., only mild dementia was mentioned.

Source	Year of publication	Country of origin	Journal abbreviation	Impact factor	Aim/ objective of study	Study design	Population					
							NH/ SNF/ LTC	NHR	Mean age (years old)	Gender		(Co)Morbidity Reason for telemedicine presentation of NHR
										m	f	
(Table S3 continued)												
Grabowski and O'Malley 2014 [131]	2014	USA	Health Aff (Millwood).	5.3	This study was designed to answer two questions. First, did the residents **of nursing homes** that were randomly chosen to receive off-hours physician coverage by a **telemedicine service** experience a lower rate of **hospitalization**, compared to residents of homes that received standard physician coverage? And second, if the nursing homes with telemedicine coverage did have lower rates of hospitalization, did they realize substantial savings?	Blinded randomized cohort study with integrated pre-post-intervention comparison and matching of NH on turnover rate.	11: 6 (I) 5 (C)	(I): 178 (C): 140	n.i.	n.i.	n.i.	n.i.; probably all cause
Hofmeyer et al. 2016 [122]	2016	USA	J Am Med Dir Assoc.	4.4	One of the main outcomes of interest in the pilot was the proportion of **residents in long term care facilities** who needed **hospital transfer** after assessment with an **electronic long-term care** provider (Avera).	pilot pre-post intervention comparison	5 in 2012 increasing to 20 in 2015	unclear: under 400 in 2012 up to over an estimated 2000 in mid-2015	n.i.	n.i.	n.i.	The chief complaints included shortness of breath (24%), skin complaint (24%), upper respiratory infection (14%), fever (13%), neurologic/syncope (12%), joint/limb pain (10%), abdominal/gastrointestinal complaint (10%), urologic (9%), weakness/dizziness (8%), and all others (32%). Among these common chief complaints, the highest proportion of transfers were for neurologic/syncope issues (66% of cases were transferred), abdominal/gastrointestinal (45%), and shortness of breath (44%). The lowest were for urologic (5%) and skin complaints (11%).
Hui et al. 2001 [132]	2001	China	Gerontology.	3.5	To assess the feasibility of **telemedicine** in providing geriatric services to **nursing home residents**, and whether this mode of care resulted in increased productivity and savings in time and costs. Outcomes: **consumption of hospital services**, e.g., outpatients, emergency department and hospital visits and duration of stay, and user satisfaction.	pilot pre-post intervention comparison	1	all residents of 1 NH: 200 beds in participating NH	n.i.	n.i.	n.i.	occupational therapist, 60% (70/117); dermatologist, 74% (55/74); podiatrist, 85% (84/99); physiotherapist, 87% (91/105); nurse, 89% (90/101); geriatrician, 95% (339/356), and psychogeriatrician, 99% (147/149).
Joseph et al. 2020 [123]	2020	USA	West J Emerg Med.	1.8	Our primary objective was to determine whether a **skilled nursing facility** based **telemedicine consultation service** staffed by emergency physicians (EP) could r**educe hospital admissions** of patients requiring acute evaluation, compared to patients who were taken directly to an ED. Our secondary objectives were to compare care escalation for conditions most amenable to on-site acute care in the SNF, and to broadly examine the financial implications of onsite acute care.	restrospective observational cohort study; broadly matching NHR on age and gender	6 (I) (C) n.i.	4606 (I): 2311 (C): 2295 broadly matched on age and gender	I: 75.6 (±12.3) C: 78.9 (±8.1)	I: 40%; C: 42%	I: 60%; C: 58%	The most common reasons for telemedicine activation were exacerbations of cardia heart failure (CHF), chronic obstructive pulmonary disease (COPD), and Diabetes mellitus (DM).

Source	Year of publica tion	Countr y of origin	Journal abbreviati on	Imp act fact or	Aim/ objective of study	Study design	Population					
							NH/ SNF/ LTC	NHR	Mean age (years old)	Gender		(Co)Morbidity Reason for telemedicine presentation of NHR
										m	f	
(Table S3 continued)												
Kane-Gill et al. 2021 [124]	2021	USA	J Am Geriatr Soc	4.2	The objective of this study was to determine the impact on incidence of high riskmedication, alert-specific ADEs, **all-cause hospitalization, 30-day readmission rates**, and consultant pharmacists' recommendations.of pharmacist-led **telemedicine** services on reducing high-risk medication adverse drug events (ADEs) for **NH residents** using medication reconciliation and prospective MRR on admission plus ongoing clinical decision support alerts throughout the residents' stay.	(quality improvement) cohort study using a stepped wedge design	4 LTC and SNF	2172	I: 77.2 (±13.6); C: 76.9 (± 13.0)	I: 35% C: 35%	I: 65%; C: 65%	Activities of daily living dependence, Alzheimer's disease, cancer, cirrhosis, deep vein thrombosis, GERD/ GI ulcer, heart failure, foot infection, osteoporosis, renal failure, schizophrenia, seizure disorder, stroke, wound infection (except foot); all comparable for intervention and control group.
Li et al. 2022 [125]	2022	USA	Health Serv Res	2.7	Independent evaluation to assess the effects of eLongTermCare (Avera eLTC Program), a **telehealth** program implemented by an integrated health system in 45 **nursing homes** across the Midwest, on the use of acute **hospital services** and total expenditures for Medicare residents.	Difference in difference design with pre-post intervention comparison; propensity score matching of NHR on demographic, health status, service use, and spending characteristics, and NH on facility characteristics (rural location, bedsize, nonprofit status, 5-star CMS rating)	45	34,228 (I): 9,608 (I; 7,194 LTC and 2,414 SNF)) (C): 24,620	80 both I and C; within I: LTC 81 and SNF 79	I: 35% C: 35%	I: 65%; C: 65%	COPD, coronary heart failure, morbid obesity, vascular disease, major depressive disorder.
Low et al. 2019 [126]	2019	Singap ore	Health Syst (Basingsto ke).	0.9-2.5	This study analyzed the economic impact of a **telegeriatrics** program on care of **nursing home residents**, from the healthcare system provider's perspective, e.g., on inpatient costs, outpatient costs, **emergency department costs, hospital admission**.	pre-post intervention comparison based on a retrospective archival data analysis of multiple data sources	4	859 consultatio ns (number of NHR n.i.)	76.2 (± 13.2)	39%	60%	Hypertension, dementia, Diabetes mellitus, hyperlipidaemia, previous stroke, depression, osteoarthritis, ischaemic heart disease, osteoporosis, previous fracture.
Lyketsos et al. 2001 [133]	2001	USA	J Geriatr Psychiatry Neurol.	2.1	To discuss employing a **videoconferencing system** in the development of a comprehensive, integrated continuum of care for Copper Ridge **SNF residents** by bridging long-term care with inpatient psychiatric care at Johns Hopkins Hospital and its impact on reducing **psychiatric hospital admissions**.	pre-post intervention comparison	1	60 bed assisted living facility and a 66 bed SNF	n.i.	n.i.	n.i.	n.i.

Source	Year of publication	Country of origin	Journal abbreviation	Impact factor	Aim/ objective of study	Study design	Population						
							NH/ SNF/ LTC	NHR	Mean age (years old)	Gender		(Co)Morbidity Reason for telemedicine presentation of NHR
										m	f	
(Table S3 continued)												
Stern et al. 2014 [129]	2014	Canada	BMC Health Serv Res.	2.0	To determine **the clinical and cost effectiveness** of enhanced multidisciplinary teams (EMDTs, via e-mail, **video or telephone link**) vs. 'usual care' for the treatment of pressure ulcers in **long term care (LTC) facilities**, with secondary outcomes being time to healing, probability of healing, pressure ulcer incidence, pressure ulcer prevalence, wound pain, **hospitalization, emergency department visits**, utility, and cost.	pragmatic cluster randomized trial using a stepped-wedge design	12	137 (I): 67 (C): 94 with 42 crossing study phases, extending from C to I i.e. double counted (MVD: probably typing error and 24 is meant)	I: 83 (±12); C: 81 (± 12)	I: 31%; C: 36%	I: 69%; C: 64%	Residents with pressure ulcer stage II or greater. *Charlson comorbidities*: Alzheimer/Dementia, Diabetes with and without end organ damage, Stroke/TIA, Para-/Hemiplegia, any solid cancer, COPD, congestive heart failure, peripheral vascular disease, myocardial infarction, moderate or severe renal disease. *Other comorbidities*: hypertension, osteoarthritis, osteoporosis, coronary artery disease, Parkinson's disease, contractures, spasticity. *Pressure ulcer risk factors*: incontinence, (urine, stool), bedbound, not alert/oriented mental status, nutritional supplement, tube feed, body mass index.
Tynan et al. 2018 [127]	2018	Australia	Aust J Rural Health.	1.5	To describe the collaboration among the health services' **Telehealth** Department, Oral Health Team and **residential aged care facilities (**RACF) staff that aimed to enable residents in rural and regional RACFs to have better access to oral health services, and to reduce the need for referral to an oral health facility (in this Australian study in cooperation with a **hospital**).	pre-post intervention comparison (quality improvement study)	4	116	n.i.	n.i.	n.i.	Oral health problems
Yeow and Huat Goh 2015 [134]	2015	Singapore	MIS Quarterly	7.2	To improve the matching of resources to patients' needs in the KGD clinic-**NH outreach program** by using **telemedicine,** thus leading to greater efficiency and reduced cost for the health-care system, e.g., improvements in organizational outcomes, such as lower **hospitalization rates**, lower uncertainty in patient wait time and better cost-managing.	pre-post intervention comparison	1-2 NH	n.i.	n.i.	n.i.	n.i.	geriatric patients classified into four specialized groups: delirium, falls, incontinence, and frailty (associated with substantial morbidity and poor health outcomes); all other geriatric patients who require general consultation are listed under general care within the clinic.

Abbreviations: NH: nursing home; LTC: long-term care facility; SNF: skilled nursing facility; NHR: nursing home resident; n.i: not indicated; I: Intervention group; C: Control group

Table S4. Included studies: Details on intervention and main findings

Source	Intervention	Facility			Duration	Service on		Evidence of the included studies on hospitalization	Evidence of the included studies on cost-effectiveness and/or health care staff's satisfaction with telemedicine use
		NH	SNF	LTC		Weekdays	After hours		
Baxter et al. 2021 [128]	Third Eye Health-Supportive Care Program: telehealth palliative care program available to nursing home residents: real-time access to a palliative care specialist to provide symptom management, goals-of-care discussions, and Medical Orders for Life-Sustaining Treatment (MOLST) completion.	X			4 months	X	X	• 0% of the telemedicine palliative care patients were hospitalized together with an increase in do-not resuscitate orders from 48% to 71% and completion of the MOLST form from 48% to 81%,<br>• 70% of the control group were hospitalized	n.i.
Catic et al. 2014 [130]	ECHO-AGE providing consultation and education by specialists to train long-term care providers to become mini geriatric medicine specialists, by advising long-term care on challenging de-identified cases with dementia and/or delirium related behavioral issues to specialists, via bimonthly 1.5 hour long secure video-conferencing. Sites are expected to engage in 8 ECHO-AGE sessions every 6 months and are encouraged to bring cases for presentation as often as they feel is needed. Emergency contact per telephone and e-mail is possible for more urgent issues that may arise between the ECHO-AGE sessions.			X	1 year	X	X	In recommendations compliant group:<br>• 74% clinically improved vs 20% in non-compliant group (P <.03, 2-sided Fisher exact test);<br>• hospitalizations less common vs non-compliant group (non-significant due to small sample size: 29% vs 60% in results; 21% vs 50% in discussion).<br>• significantly lower mortality in the 29 cases in whom improvement was reported vs the 10 cases who did not improve (4% vs 50%, P < .003, 2-sided Fisher exact test).	n.i.
Chess et al. 2018 [119]	Integration of TripleCare (TC) into a SNF. TC is a physician group specialized in caring for medically frail patients through telemedicine (mobile device operational at patient's bedside for video conference to interview the patient). When possible: physical exam using the unit's digital stethoscope and 18x zoom camera. Integration of TripleCare (TC) in after-hour emergency situations. The Tmed physicain informed the attending physician immediately or the next day depending on necessity.		X		1 year		X	18%* reduction in hospitalization compared to the year before the intervention started. Comment MVD: ca. 12% when occupancy rates are taken into account (50% overestimation of published hospitalization rate)	Under assumptions: ca. USD 1.6 million in this 1 SNF during this period; USD 4167 per bed/year saved on Medicare expenses. Taking everything in account (annual service expenses USD 60,000; decreased transportation costs; capturing lost Medicaid days) it is estimated that the facility netted USD 20,000 above the cost of the program.
Dadosky et al. 2018 [120]	The setting was after discharge from a tertiary acute-care hospital to a suburban post-acute SNF (and eventually home with home healthcare). For up to 30 days following hospital discharge, patients received interactive telemanagement care, which included video sessions, POC laboratory testing, remote auscultation with stethoscope, and vital sign monitoring from HF clinicians, uploaded and continuously visible to SNF staff on the Zephyr dashboard system. Sessions were planned as needed according to the change in patient condition, prioritized by stratification via the dashboard system		X		21 months, individual follow-up= 30 days	X	X	Reduction in rehospitalization rates: telemanagement group 17% to 24% historical controls: absolute risk reduction of 6.51% and relative risk reduction of 27.24% within 30 days post hospital discharge, despite higher predicted rehospitalization risk.<br>Higher predicted rehospitalization risk may have contributed to the telemanagement hospitalization rate not being significantly lower than that of the control group.	The overall cost for telehealth equipment was USD 1,386.00 per patient or USD 30.24 per patient per day. Hospital savings was estimated at USD 9,234.54 based on the institution's readmission cost.

Source	Intervention	Facility			Duration	Service on		Evidence of the included studies on hospitalization	Evidence of the included studies on cost-effectiveness and/or health care staff's satisfaction with telemedicine use
		NH	SNF	LTC		Weekdays	After hours		
(Table S4 continued)									
De Luca et al. 2016 [121]	Sicilian Tele-Health-Care Model intervention: Monitoring of patient's vital signs three times a week plus weekly video-consultation either by a neurological or a psychologist, ensuring assistance continuity with a multi-parametric vital sign monitoring, and periodic neurological and psychological tele-consulting compared to control group with standard in -home nursing care; 3x/week; vital signs monitoring; weekly consultation by neurologist or a psychologist.	X			n.i. just mentioning base-line (t0) and follow-up (t1)	X		Admission to health care services in telemonitoring group 3/32 (9%) vs controls 8/27 (30%; Chi^2-test $p<0.05$).	n.i.
Grabowski and O'Malley 2014 [131]	Pre-intervention health care in off-hours provided by telephone by covering physician in group practice from a remote location.<br>Intervention: a cart with equipment for two-way videoconferencing and a high-resolution camera for use in wound care. When a nursing home resident had an off-hours medical problem, a staff member brought the cart into the resident's room and contacted the telemedicine service.	X			2 years: 13 months pre-intervention, 11 months post-intervention		X	The raw rate of hospitalizations declined 5.3 percent for the control group and 9.7 percent for the treatment group. Thus, the pre-post difference in hospitalizations in the treatment group was 4.4 percentage points lower than the pre-post difference in the control group. This decline was only significant in the hospitalization rate at more-engaged facilities. The label more engaged NH facility depended on frequency and types of telemedicine calls by month and facility. A NH that typically had 180 hospitalizations per year and that was more engaged with telemedicine could expect to see a statistical significant reduction of about 15.1 hospitalizations each year, relative to a NH that was less engaged.	The average savings to Medicare for a nursing home that was more engaged with telemedicine would be USD 151,000 per nursing home per year, relative to the less-engaged facilities. The annual cost of the telemedicine service in this study was USD 30,000 per nursing home, implying net savings of roughly USD 120,000 per nursing home per year in the more-engaged facilities.
Hofmeyer et al. 2016 [122]	Avera eLTC Program: 2-way video communication and peripherals; allowing real-time communication between residents and providers in facilities with on-call specialists. Specialty equipment, such as a 2-way stethoscope and high-definition camera, allowed providers to listen to lungs, heart, and abdomen at a distance, as well as gaining a closer view of the patient as needed; including upgrade of infrastructure in facilities when necessary.			X	3 years	X	X	The telemedicine service (eLTC) was offered for patients that normally all would have been hospitalized. The longer eLTC is offered, the lower the rate of transfers of eLTC becomes:<br>• in first two years hospital transfer rate of eLTC consultations 39% and 54%, respectively;<br>• in last two years 29% and 17%, respectively.	n.i.
Hui et al. 2001 [132]	Community Geriatric Assessment Teams (CGAT) plus dermatology and podiatry services per real-time, two-way audio-video-teleconferencing with high resolution camera at NH), used to replace conventional geriatric outreach services for routine follow-up as well as acute deterioration requiring urgent review. Face-to-face consultation only in cases where telemedicine alone seemed inadequate for assessment or management of the resident..	X			1 year	X		• 10.6% reduction in admissions to an acute hospital (pre-intervention year compared to the post-intervention (study) year), with a drop of 9.6% in total length of stay.<br>• emergency department visits decreased by 8.8% (pre-post intervention comparison), even though 55% of all AED visits occurred in Tmed non-available after office hours<br>• Admission to a convalescent hospital rose with 19% and the total bed days with 20%	• Telemedicine was cheaper than outreach or clinic visits for all participating disciplines.<br>Total cost savings for 1 NH in 1 year:<br>• hospitalization of USD 30,510<br>• transport and escort services to outpatient clinics and emergency department of USD 9,510<br>• Calculations on productivity gains and savings are not published in this article.<br>• Generally positive attitude of NH staff towards telemedicine, though 50% felt that their workload increased; diverse views on image and sound quality.

Source	Intervention	Facility			Duration	Service on		Evidence of the included studies on hospitalization	Evidence of the included studies on cost-effectiveness and/or health care staff's satisfaction with telemedicine use
		NH	SNF	LTC		Weekdays	After hours		
(Table S4 continued)									
Joseph et al. 2020 [123]	SNF-based on-demand telemedicine services facilitated by a clinical care specialist (CCS) wo is a paramedic or emergency medical technician on-site at all times. The service is used for acute evaluations when facility staff judged that patients would otherwise require ED transfer. The CCS uses a cart with point-of-care labs, electrocardiograms, telemetry, and ultrasound. Patients can also be directly transported for outpatient imaging. (X-Ray/ computed tomography). Order sets and pathways are used to streamline decisions to treat in place or transfer. The CCS monitors SNF residents in accordance with emergency physician orders and can re-initiate consultations. Controls received traditional ED-based care.		X		1 year	X	X	27% of the SNF-based group were transferred to the ED, whereas 71% of the control group were admitted to the hospital from the ED (OR = 0.15 (95% CI, 0.13-0.17), p < 0.001). These results were directionally consistent across the top three conditions (CHF, COPD, DM), although rates of presentation for all three were significantly higher in the SNF based<br>group	The average cost of the telemedicine service in this study was USD 816 per episode, compared to the flat rate of USD 30,000 per facility per year charged by Grabowski et al. [6]. Amortized across 2311 consultations in six SNFs over a one-year period, this represents a more than tenfold increase. Conversely, the average Medicare payment for a SNF-based rehospitalization is over USD 10,000.30. Considering the added expenses of ambulance transportation and EP fees, this enhanced telemedicine service would be cost-effective if it averted 10% of hospitalizations. The data from this program suggests an 80% reduction in care escalation, suggesting this is a worthwhile investment, irrespective of the clinical benefits from avoiding unnecessary admissions.
Kane-Gill et al. 2021 [124]	Pre- and post-NH-admission intervention components using video-tablet-based telemedicine by pharmacist:<br>At LTC/SNF (re)admission medication reconciliation with cognitive intact residents. In case of high-risk medication irrespective of cognitive function: prospective Medication Regimen Review (MRR) within ca. 72-hours of admission. Communication to provider on potential medication-related problems. Resident-pharmacist telemedicine interactions also included educating the resident about ADE-related symptoms and early detection.<br>Post-admission, pharmacists received clinical decision support alerts that were formulated within an electronic health record (EHR)-agnostic platform (TheraDoc) to detect potentially inappropriate prescribing. Alerts were used to prevent ADE by intervening. Alerts were delivered to the CP (consultant pharmacist) in real-time and were managed during normal weekday working hours. Resident-pharmacist telemedicine interactions were conducted within approximately 72 hours of CP alert receipt, when deemed to be clinically necessary.<br>Usual care included federally-mandated monthly retrospective MRRs and on demand MRRs at the clinician's request.		X	X	1 year, intervention data of the last 9 months	X		Group comparison (I) vs (C):<br>• All cause hospitalization was similar (I) 149 vs (C) 138; 2.33 vs 2.70/1,000-resident-days; AIRR = 1.06 (95% CI = 0.72–1.58); P = .754),<br>• 30-day readmissions (110 vs 102; 1.72 vs 2.00/1,000-resident-days; AIRR = 1.21 (95% CI = 0.76–1.93); P = .422).<br>• Sensitivity analysis with a Poisson model did not meaningfully alter these results.	The physician acceptance rate was 57.1%; after adjusting the denominator for no responses and discharged residents, the acceptance rate was 66.5%.

Source	Intervention	Facility			Duration	Service on		Evidence of the included studies on hospitalization	Evidence of the included studies on cost-effectiveness and/or health care staff's satisfaction with telemedicine use
		NH	SNF	LTC		Weekdays	After hours		
(Table S4 continued)									
Li et al. 2022 [125]	Avera eLTC Program: a telehealth program implemented by an integrated health system:<br>The eLTC program included three components:<br>1. Telehealth consults for urgent and specialty care provided 24/7 by a team for all nursing home residents. Evaluation of resident via synchronous two-way audio and video, if necessary, and instruction on care plan for LTC staff.<br>2. Telehealth transitional care coordination, categorizing residents in high and low risk categories for ED and hospitalization, with detailed care plans for both categories and additional schedule for telemedicine sessions for high risk residents and medication review for low risk residents.<br>3. Staff training and empowerment.		X	X	40 months of enrollment data:<br>C: 12 months<br>I: 24 months follow-up	X	X	For LTC residents:<br>• Estimated reduction of 73 ED visits per 1000 beneficiaries (p < 0.01), 8.6% reduction over the two-year follow-up period.<br>• Non significant decline in number of hospitalizations for the full group<br>• Significant decline in number of hospitalization for sub-group of newly admitted LTC-residents:<br>• ED visits: 237/1000 NHR/2 years of follow-up (p<0.01, 18.5% effect)<br>• Hospitalizations: 84/1000 NHR/2 years of follow-up (p<0.01, 6.1% effect)<br>• For SNF-residents:<br>• ED visits: 85/1000 NHR/2 years of follow-up (p=0.03, 9.7% effect)<br>• No discernable change in the number of hospitalizations and likelihood of 30-day readmission.	For LTC-residents:<br>• In the full group: marginally significant reduction of Medicare expenditures by USD 73 /resident/month (p = 0.08) over 2 years, which represents a 3.6% reduction for the full group.<br>• For newly admitted LTC-residents: significant reductions in total Medicare expenditures by USD 467 /resident/month (p<0.01, 11.3% effect).<br>For SNF residents:<br>• No statistically significant reduction in total Medicare expenditures.<br>Avera Health reported that it would take a one-time fee of USD 250 plus a monthly fee of USD 55 per resident to cover the cost of the eLTC program (regardless of the frequency of telehealth consults provided). Thus the program would generate a net saving of USD 5 PBPM, amounting to a total saving of USD 95 per long-term care resident over the average stay.
Low et al. 2019 [126]	GeriCare@North: Remote video consultation between partnered NH and a hospital in 4 years: A Polycom videoconferencing system/set (telegeriatrics) incorporated a hub and spoke model and was used to conduct teleconsultations, multidisciplinary team meetings, mortality audits and continuous nursing education with the staff of the four participating nursing homes. A high-resolution camera and high-definition video monitor were installed in the acute hospital and each nursing home (information exchange by encrypted high-speed internet).	X			4.5 years:<br>C: first half year<br>I: last 4 years	n.i.	n.i.	• Emergency department transfers per 100,000 resident days decreased from 158 in Year 1 to 37 in Year 5.<br>• Hospital admission rate was 159 per 100,000 resident days in Year 1 and 51 in Year 5.	Under assumptions: after introduction of teleconsultations, all the three costs – specialist outpatient (significant cost reduction USD 83.366 per month), inpatient (significant cost reduction USD 470.971 per month) and ED (non-significant reduction) – reduced over time. With every unit increase in the number of teleconsultation sessions, the specialist outpatient, inpatient and emergency department costs declined.<br>However, for every percentage increase in staff trained for the month there is a corresponding decline of USD 39.546 in emergency department costs
Lyketsos et al. 2001 [133]	Device: "Three-decker" cart with electrical wiring on wheels, 12-inch television, high-speakerphone and an 8x8-brand videoconferencing camera. Twice weekly routine calls to discuss residents recently/ currently/ or about to be hospitalized. The purpose of each conference was to update the remote team of the patient's status, review current care needs, develop a joint care plan, and discuss follow-up and continuity of care issues.		X		2 years:<br>C: first year<br>I: last year	X		Comparing 1998 to 1999 data: an almost 50% decline in the number of hospitalizations (from 21 to 11) . During this period of time, there were also 100 fewer days spent in the hospital (from 367 to 258). However, the mean length of stay increased by almost 6 days (from 17.4-23.5), suggesting that only the most impaired residents were now being admitted to the hospital.	The telemedicine program provided improved fine-tuning and understanding between facility and hospital, so that residents were more successfully reintegrated to the facility on return from the hospital. Clinical teams in different organizations became part of a whole comprising an effective continuum of care and brought about cost-effective positive outcomes for residents, families and staff. All in acquisition of equipment costs of USD 2375.

Source	Intervention	Facility			Duration	Service on		Evidence of the included studies on hospitalization	Evidence of the included studies on cost-effectiveness and/or health care staff's satisfaction with telemedicine use
		NH	SNF	LTC		Weekdays	After hours		
(Table S4 continued)									
Stern et al. 2014 [129]	Intervention in two phases: Phase 1 (3 months in length) focused on education of each LTC staff by APNs on 1d/week to establish multidisciplinary wound care teams within LTC. One wound care lead was primary contact for the advanced practice nurse (APN). Digital camera for wound picturing and assessment.<br>Phase 2 (1-11 months in length): remote support of the facility wound care lead by the APNs based on de-identified resident data and wound pictures by e-mail and telephone. Face-to-face/video-consultation of the expert team on an off-site hospital if required. APN visited LTCs when needed/ on request. This process was repeated every 2 weeks for all PUs until healed, or until the end of the study period.<br>Control "ususal wound care" eventually with/ without expertise in wound care.			X	17-18 months	X		No statistically significant differences were found regarding hospitalization and ED visits utility. Mean hospitalization rate was estimated to be 1.2 (p = 0.59) times larger during the intervention period than during the control period. ED visit rate was estimated to be 1.3 (p = 0.52) times larger during the intervention period than during the control period.<br>Effective uptake of the intervention across most facilities was prohibited by inadequate allociation of staff time to its implementation, unavailability of the required wound care supplies and diverse implementation of the intervention between facilities.	Costs: Intervention teams were estimated to reduce direct care costs of on average USD 649 per resident compared to 'usual care', onsite support by APN wound specialists was welcomed, and is responsible for reduced costs.<br>Effective uptake of the intervention across most facilities was prohibited by inadequate allociation of staff time to its implementation, unavailability of the required wound care supplies and diverse implementation of the intervention between facilities.
Tynan et al. 2018 [127]	Cooperation between Teledentistry Health Service, Oral Health Team and Residential Aged Care Facility (NH) session by live video-conferencing, if required after a visit by an oral health therapist (OHT) for screening, simple intervention. The teledentistry appointment involved the OHT using an intraoral camera probe to transmit a live feed of the resident's mouth to a dentist (still images were captured for later review) who was located at another health care facility. Each NH also had a nurse allocated to the oral health portfolio to ensure compliance with the oral health accreditation and to be trained specifically by the OHT to disseminate knowledge to other NH staff.	X			6 months	X		Access to teledentistry after screening prevented 14 residents (12%) from the need to travel to an oral health facility.	Staff members/ managers: increased awareness of residents' oral health needs and prevention requirements; improved access to resources for oral health management; minimizing disruption to residents. Facility managers: positive cultural change in staff towards oral health care, attributed to education and access to oral health specialists on site more regularly. Initial screenings result in earlier detection of health problems.<br>Display of costs in AUD per appointment for three scenarios: OHT screening at NH was the least cost scenario, the second was teledentistry at NH, and the third attendance at oral health clinic via car/ ambulance. Costs of Teledentistry are given, but no all-inclusive cost comparison.
Yeow and Huat Goh 2015 [134]	Telemedicine using Polycom Videoconferencing system to support geriatric patients who reside in nursing homes within its health cluster, also by streamlining the flow of NH patients by a more flexible clinical pathway, allocating patients more efficiently to the most appropriate hospital physician choosing out of a larger pool of physicians for virtual consultation..	X			11 months:<br>C: first 3 months<br>I: last 8 months	X	X	Reduction in ED visits and hospitalization.	Reallocation of care and new clinical pathways were ambiguous on cost-effectiveness: a switch from senior specialist to registrar physician care made health care cheaper, but the registrar needed more time per NHR, which made it more expensive again. Also the registrar is not apt for more challenging cases. The senior physician doing more consults on request of the NHR caused higher expenses.

Abbreviations: NH: nursing home; NHR: nursing home resident; SNF: skilled nursing facility; LTC: long-term care facility; ED: Emergency Department; APN: Advanced Practice Nurse; Tmed: telemedicine; I:intervention group; C: control group; PU: Pressure Ulcer; n.i.: not indicated; n.a.: not applicable; MVD: first author; USD: United States Dollar/s

References of included studies

[128] Baxter, K. E.; Kochar, S.; Williams, C.; Blackman, C.; Himmelvo, J., Development of a Palliative Telehealth Pilot to Meet the Needs of the Nursing Home Population. J Hosp Palliat Nurs 2021, 23, (5), 478-483; DOI: 10.1097/njh.0000000000000784.

[130] Catic, A. G.; Mattison, M. L.; Bakaev, I.; Morgan, M.; Monti, S. M.; Lipsitz, L., ECHO-AGE: an innovative model of geriatric care for long-term care residents with dementia and behavioral issues. J Am Med Dir Assoc 2014, 15, (12), 938-42; DOI: 10.1016/j.jamda.2014.08.014.

[119] Chess, D.; Whitman, J. J.; Croll, D.; Stefanacci, R., Impact of after-hours telemedicine on hospitalizations in a skilled nursing facility. Am J Manag Care 2018, 24, (8), 385-388.

[120] Dadosky, A.; Overbeck, H.; Barbetta, L.; Bertke, K.; Corl, M.; Daly, K.; Hiles, N.; Rector, N.; Chung, E.; Menon, S., Telemanagement of Heart Failure Patients Across the Post-Acute Care Continuum. Telemed J E Health 2018, 24, (5), 360-366; DOI: 10.1089/tmj.2017.0058.

[121] De Luca, R.; Bramanti, A.; De Cola, M. C.; Trifiletti, A.; Tomasello, P.; Torrisi, M.; Reitano, S.; Leo, A.; Bramanti, P.; Calabrò, R. S., Tele-health-care in the elderly living in nursing home: the first Sicilian multimodal approach. Aging Clin Exp Res 2016, 28, (4), 753-9; DOI: 10.1007/s40520-015-0463-8.

[131] Grabowski, D. C.; O'Malley, A. J., Use of telemedicine can reduce hospitalizations of nursing home residents and generate savings for medicare. Health Aff (Millwood) 2014, 33, (2), 244-50; DOI: 10.1377/hlthaff.2013.0922.

[122] Hofmeyer, J.; Leider, J. P.; Satorius, J.; Tanenbaum, E.; Basel, D.; Knudson, A., Implementation of Telemedicine Consultation to Assess Unplanned Transfers in Rural Long-Term Care Facilities, 2012–2015: A Pilot Study. Journal of the American Medical Directors Association 2016, 17, (11), 1006-1010; DOI: 10.1016/j.jamda.2016.06.014.

[132] Hui, E.; Woo, J.; Hjelm, M.; Zhang, Y. T.; Tsui, H. T., Telemedicine: a pilot study in nursing home residents. Gerontology 2001, 47, (2), 82-7; DOI: 10.1159/000052778.

[123] Joseph, J. W.; Kennedy, M.; Nathanson, L. A.; Wardlow, L.; Crowley, C.; Stuck, A., Reducing Emergency Department Transfers from Skilled Nursing Facilities Through an Emergency Physician Telemedicine Service. West J Emerg Med 2020, 21, (6), 205-209; DOI: 10.5811/westjem.2020.7.46295.

[124] Kane-Gill, S. L.; Wong, A.; Culley, C. M.; Perera, S.; Reynolds, M. D.; Handler, S. M.; Kellum, J. A.; Aspinall, M. B.; Pellett, M. E.; Long, K. E.; Nace, D. A.; Boyce, R. D., Transforming the Medication Regimen Review Process Using Telemedicine to Prevent Adverse Events. J Am Geriatr Soc 2021, 69, (2), 530-538; DOI: 10.1111/jgs.16946.

[125] Li, S. E.; Hossain, M.; Gilman, B.; Forrow, L. V.; Lee, K. M.; Brown, R., Effects of a nursing home telehealth program on spending and utilization for Medicare residents. Health Serv Res 2022, 1-10; DOI: 10.1111/1475-6773.13936.

[126] Low, J. A.; Hui Jin, T.; Tan Lean Chin, L.; Agarwal, N.; Kim Huat, G.; Yeow, A.; Soh, C., Cost analysis of implementing a telegeriatrics ecosystem with nursing homes: panel data analysis. Health Syst (Basingstoke) 2019, 9, (4), 285-292; DOI: 10.1080/20476965.2019.1589390.

[133] Lyketsos, C. G.; Roques, C.; Hovanec, L.; Jones, B. N., 3rd, Telemedicine use and the reduction of psychiatric admissions from a long-term care facility. J Geriatr Psychiatry Neurol 2001, 14, (2), 76-9; DOI: 10.1177/089198870101400206.

[129] Stern, A.; Mitsakakis, N.; Paulden, M.; Alibhai, S.; Wong, J.; Tomlinson, G.; Brooker, A. S.; Krahn, M.; Zwarenstein, M., Pressure ulcer multidisciplinary teams via telemedicine: a pragmatic cluster randomized stepped wedge trial in long term care. BMC Health Serv Res 2014, 14, 83; DOI: 10.1186/1472-6963-14-83.

[127] Tynan, A.; Deeth, L.; McKenzie, D.; Bourke, C.; Stenhouse, S.; Pitt, J.; Linneman, H., Integrated approach to oral health in aged care facilities using oral health practitioners and teledentistry in rural Queensland. Aust J Rural Health 2018, 26, 290-294; DOI: 10.1111/ajr.12410.

[134] Yeow, A.; Goh, K. H., Work Harder or Work Smarter? Information Technology and Resource Allocation in Healthcare Processes. MIS Quarterly 2015, 39, (4), 763-786.
